# Macrophage lipid metabolism and inflammasome activation in rheumatoid arthritis: emerging mechanisms and therapeutic implications

**DOI:** 10.3389/fimmu.2026.1902920

**Published:** 2026-10-14

**Authors:** Hua Hao, Hongyu Zhao, Ping Dong, Xuanting Fan, Jun Liu, Hao Wu, Lifen Jia, Chen Li, Limei Ao, Qiumei Dong

**Affiliations:** 1 College of Traditional Chinese Medicine, Inner Mongolia Medical University, Hohhot, China; 2 Department of Spleen and Stomach Diseases, The Traditional Chinese and Mongolian Medicine Hospital of Hohhot, Hohhot, China

**Keywords:** fatty acid synthase, lipid metabolism, liver X receptor, macrophages, NLRP3 inflammasome, palmitoylation, peroxisome proliferator-activated receptor, rheumatoid arthritis

## Abstract

Rheumatoid arthritis (RA) is a chronic autoimmune disease characterized by persistent synovial inflammation, synovial hyperplasia, cartilage destruction, and bone erosion. Within the multicellular RA synovial network, macrophages act as important inflammatory effectors and metabolic sensors whose functional states are shaped by lipid metabolic reprogramming. In RA synovium, impaired cholesterol efflux and altered fatty acid flux remodel membrane lipid composition and lipid-raft-dependent Toll-like receptor (TLR)–NF-κB signaling, thereby enhancing the expression of NOD-like receptor family pyrin domain-containing 3 (NLRP3) and pro-interleukin-1β (pro-IL-1β). Cholesterol overload or crystallization, saturated fatty acid-induced lipotoxicity, mitochondrial and lysosomal injury, mitochondrial reactive oxygen species, ionic imbalance, and lipid peroxidation generate activation-associated signals that lower the threshold for NLRP3 inflammasome activation. Mechanistic studies, largely outside RA, suggest that fatty acid synthase (FASN)-dependent production of palmitate-derived acyl donors may support ZDHHC-mediated NLRP3 palmitoylation, subcellular trafficking, NEK7 interaction, ASC recruitment, and inflammasome assembly. Liver X receptor (LXR) and peroxisome proliferator-activated receptor (PPAR) function as complementary lipid-sensing regulators: LXR mainly controls cholesterol efflux and membrane signaling, whereas PPARs coordinate fatty acid handling, mitochondrial adaptation, efferocytosis, and resolution-associated programs. Activation of the NLRP3–caspase-1 axis promotes IL-1β and IL-18 maturation and gasdermin D-mediated pyroptosis, amplifying macrophage–fibroblast-like synoviocyte crosstalk, immune-cell recruitment, osteoclastogenesis, and structural joint damage. Because direct validation of the complete FASN–palmitoylation–NLRP3 sequence in RA synovial macrophages remains limited, this axis is presented as a testable RA-relevant mechanistic framework rather than an established RA-specific pathway while highlighting potential targets for macrophage-directed immunometabolic intervention.

## 1 Introduction

Rheumatoid arthritis (RA) is a chronic autoimmune disease characterized by persistent synovial inflammation, abnormal synovial hyperplasia, cartilage destruction, and bone erosion. As the disease progresses, patients may develop joint swelling and pain, morning stiffness, restricted mobility, and even irreversible joint deformity, which substantially impairs quality of life (1). For many years, research on RA pathogenesis has focused mainly on autoantibody formation, aberrant activation of T and B cells, and sustained release of inflammatory cytokines such as tumor necrosis factor-α (TNF-α), interleukin-1β (IL-1β), and interleukin-6 (IL-6). Increasing evidence from immunometabolism, however, indicates that RA is not driven solely by immune-inflammatory signaling. Metabolic reprogramming of immune cells in the synovial microenvironment also provides a fundamental basis for persistent inflammation, tissue invasion, and bone destruction (2). In RA synovial lesions, macrophages represent one of the key effector populations linking innate immunity, adaptive immunity, and tissue destruction. The abundance and activation status of synovial macrophages correlate closely with disease activity, local cytokine levels, and structural joint damage. Activated macrophages secrete TNF-α, IL-1β, IL-6, and multiple chemokines, thereby contributing to inflammatory amplification, immune-cell recruitment, and angiogenesis. They also sustain the synovial inflammatory network through interactions with fibroblast-like synoviocytes (FLS), T cells, B cells, and osteoclast precursors (3, 4). In the inflammatory synovial microenvironment, macrophages do not remain in a fixed M1/M2 binary state. They exhibit marked heterogeneity and plasticity instead, and their functional states are shaped jointly by cytokines, hypoxia, metabolic substrates, lipid mediators, and cell–cell interactions.

Lipid metabolic reprogramming provides a useful entry point for understanding sustained macrophage activation in RA. Fatty acid uptake, *de novo* fatty acid synthesis, fatty acid oxidation (FAO), cholesterol efflux, membrane lipid remodeling, and lipid mediator production not only supply energy and materials for membrane renewal but also directly regulate macrophage functional states, inflammatory signal transduction, and inflammation resolution—for example, cholesterol accumulation can alter lipid raft architecture and enhance Toll-like receptor (TLR)-associated inflammatory signaling, whereas saturated fatty acids and lipotoxic stress can induce mitochondrial dysfunction, oxidative stress, and endoplasmic reticulum (ER) stress (5, 6). Conversely, enhanced FAO and production of pro-resolving lipid mediators are closely linked to termination of inflammation, tissue repair, and restoration of immune homeostasis (7). Thus, lipid metabolism is not a passive background process in macrophage inflammatory responses; it is a regulatory layer that helps determine inflammatory fate and functional outcome.

Lipid metabolic abnormalities can also convert metabolic stress into inflammatory output through inflammasome activation. The NOD-like receptor family pyrin domain-containing 3 (NLRP3) inflammasome senses danger signals such as cholesterol crystals, lipid peroxidation, mitochondrial reactive oxygen species (mtROS), and organelle damage. It promotes caspase-1 activation, maturation, release of IL-1β and IL-18, and pyroptosis, thereby aggravating synovial inflammation and tissue injury (8). At the same time, liver X receptor (LXR) and peroxisome proliferator-activated receptor (PPAR), as lipid-sensing nuclear receptors, modulate macrophage function by regulating cholesterol homeostasis, fatty acid utilization, and inflammatory gene transcription (9). *De novo* fatty acid synthesis mediated by fatty acid synthase (FASN), together with protein palmitoylation, provides emerging mechanistic clues for how lipid synthesis may influence NLRP3 inflammasome assembly (10). Importantly, these lipid-metabolic mechanisms operate within a disease-specific synovial niche rather than in isolation. RA synovium combines persistent autoantibody and immune-complex signaling, hypoxia and lactate accumulation, invasive RA-FLS activity, continuous monocyte influx, and osteoclastogenic cues at the synovium–bone interface (11, 12). The spatial convergence of these factors across the synovial lining, sublining, perivascular regions, and bone-adjacent areas generates macrophage states associated with active inflammation, remission, or structural joint damage. Accordingly, evidence obtained from human RA synovium or synovial fluid and from experimental arthritis models is distinguished from mechanistic evidence derived from non-RA inflammatory systems.

This review focuses on how lipid metabolic reprogramming links RA synovial macrophage activation to NLRP3 inflammasome signaling. Impaired cholesterol efflux and altered fatty acid flux reshape membrane lipid composition and lipid-raft-dependent TLR–NF-κB signaling, thereby enhancing the expression of NLRP3 and pro-IL-1β. Cholesterol accumulation or crystallization, saturated fatty acid-induced lipotoxicity, mitochondrial dysfunction, lysosomal injury, and lipid peroxidation generate organelle stress and danger signals that promote NLRP3 activation. FASN-dependent production of palmitate and palmitoyl-CoA may support NLRP3 palmitoylation, subcellular trafficking, NEK7 binding, ASC recruitment, and inflammasome assembly. LXR and PPAR regulate these processes through their effects on cholesterol homeostasis, fatty acid handling, and mitochondrial function. Activation of the NLRP3–caspase-1 axis promotes IL-1β and IL-18 maturation and GSDMD-mediated pyroptosis, thereby amplifying macrophage–FLS crosstalk, immune-cell recruitment, osteoclastogenesis, and persistent synovial inflammation in RA. This review integrates cholesterol handling, fatty acid flux, LXR/PPAR signaling, FASN-dependent palmitoylation, NLRP3 priming, activation, trafficking, and assembly into a continuous mechanistic framework centered on RA synovial macrophages. These processes are discussed not as isolated metabolic or inflammatory modules but as sequential and context-dependent events within the multicellular RA synovial microenvironment, with particular attention to their potential therapeutic implications. Relevant literature was searched in PubMed and Web of Science Core Collection from database inception to July 2026. The search terms included combinations of “rheumatoid arthritis,” “synovial macrophage,” “lipid metabolism,” “immunometabolism,” “fatty acid metabolism,” “cholesterol metabolism,” “LXR,” “PPAR,” “FASN,” “palmitoylation,” “NLRP3 inflammasome,” “lipidomics,” and “metabolomics.” The reference lists of relevant articles were also reviewed to identify additional studies.

## 2 Macrophages in RA synovial inflammation: inflammatory effectors and metabolic sensors

### 2.1 Heterogeneity of RA synovial macrophages

Macrophages in RA synovial tissue are not a single homogeneous inflammatory cell population. They comprise subpopulations with distinct origins, spatial distributions, and functional states (13). Recent studies have increasingly recognized that RA synovial macrophages include both tissue-resident macrophages that persist in synovial tissue and help maintain tissue homeostasis as well as infiltrating monocyte/macrophage populations derived from peripheral blood monocytes recruited into the synovium (13–15). These cells are distributed differently in the synovial lining, sublining layer, perivascular regions, and inflammatory cell aggregates, and they contribute to inflammatory amplification, antigen presentation, chemokine release, efferocytosis, tissue repair, and inflammation resolution.

Spatially, macrophages in the synovial lining are closer to the joint cavity and synovial fluid. They can participate in local joint homeostasis by phagocytosing cellular debris, clearing apoptotic cells, and maintaining a barrier-like tissue structure (16). Some macrophage subsets associated with MERTK, TREM2, or LYVE1 are considered to have homeostatic, immunoregulatory, and pro-resolving features and may contribute to remission in RA (17). In contrast, active RA synovium often contains abundant infiltrating monocyte/macrophage populations, which generally show stronger inflammatory transcriptional programs (18).

RA synovial macrophages, therefore, cannot be adequately described by the traditional M1/M2 dichotomy. Although the M1/M2 framework remains useful for broadly distinguishing inflammatory from repair- or resolution-associated functions, single-cell studies have revealed a substantially more complex organization of macrophage states in RA synovium. Inflammatory IL1B-, FCN1-, HBEGF-, S100A12-, SPP1-, and interferon-response-associated programs coexist with MERTK-, TREM2-, and LYVE1-associated programs related to phagocytosis, tissue homeostasis, efferocytosis, and remission (13, 18). These transcriptional and functional programs are partially overlapping rather than mutually exclusive, and individual macrophage populations may acquire mixed features in response to local environmental cues. Their distribution is further influenced by cellular origin, anatomical niche, cytokines, immune complexes, hypoxia, metabolic substrates, lipid mediators, extracellular matrix components, and neighboring FLS. RA synovial macrophages are, therefore, best understood as a collection of context-dependent, disease-associated functional states arising from tissue-resident and infiltrating monocyte-derived populations rather than as a single specialized phenotype separate from the conventional M1/M2 classification. Across different disease stages, tissue regions, and treatment contexts, these macrophage states may shift among inflammatory amplification, tissue destruction, immune regulation, and inflammation resolution, contributing to the persistence and heterogeneity of RA synovitis. Inflammatory macrophages are major sources of TNF-α, IL-1β, and multiple chemokines and directly promote persistent synovial inflammation. Conversely, dysfunction of homeostatic or repair-associated macrophages may weaken inflammation resolution, efferocytosis, and restoration of tissue homeostasis. RA synovial macrophages are, therefore, neither merely terminal inflammatory effector cells nor fixed immune-cell subtypes. They are dynamic cell populations that sense changes in the synovial microenvironment and adapt metabolically and functionally. To facilitate comparison of the origins, localization, and functional features of different macrophage subsets in RA synovium, the major macrophage states and their relationships with inflammatory activity or remission in RA are summarized in **Table 1**.

**Table 1:** Major macrophage subpopulations in RA synovium and their functional features.

Subpopulation/state	Main origin or localization	Representative markers	Major functions	Relationship with RA pathology or remission	References
Tissue-resident synovial macrophages	Embryonic origin or local self-renewal; mainly located in the synovial lining	CX3CR1, MERTK, TREM2, LYVE1, etc.	Maintain the synovial barrier, clear cellular debris, and restrict inflammatory spread	Contribute to local joint homeostasis; functional impairment may promote persistent inflammation	(13, 14)
MERTK+TREM2+ macrophages	Mostly associated with homeostatic or remission-phase synovium	MERTK, TREM2, CD206	Efferocytosis, immune regulation, lipid handling, and inflammation resolution	Relatively enriched during RA remission and may participate in termination of inflammation and restoration of synovial homeostasis	(13, 17)
MERTK+LYVE1+ macrophages	Homeostatic synovium or perivascular regions	MERTK, LYVE1, CD206	Tissue repair, matrix homeostasis, and inflammatory restraint	Associated with synovial homeostasis, treatment response, or remission, and may reflect a protective macrophage network	(13, 16)
Infiltrating monocyte/macrophage populations	Derived from peripheral blood monocytes recruited into the synovium; commonly found in the sublining of inflamed synovium	CCR2, CD14, CD16, CD48, etc.	Release of pro-inflammatory cytokines, chemokine production, and immune-cell recruitment	Expanded in active RA and represent an important cellular source of synovial inflammatory amplification and tissue destruction	(13, 19)
Inflammatory myeloid cells/macrophages	Locally expanded in inflamed synovium or differentiated from infiltrating monocytes	IL1B, S100A12, SPP1, TNF, etc.	Secrete IL-1β, TNF-α, chemokines, and tissue destruction-related molecules	Closely associated with RA synovial inflammatory activity, cellular infiltration, and tissue invasion	(18, 19)
Osteoclast-related monocyte/macrophage precursors	Monocyte/macrophage lineage; mainly enriched at the synovium-bone interface	RANK, CSF1R, NFATc1, CTSK, etc.	Differentiate into osteoclasts and mediate bone resorption	Directly link synovial inflammation to bone erosion and form an important cellular basis of RA bone destruction	(20, 21)

### 2.2 Macrophage-derived inflammatory mediators and RA progression

In the RA synovial microenvironment, the importance of macrophages is reflected not only by their increased abundance and phenotypic changes but also by their sustained release of inflammatory mediators, which generates inflammatory amplification loops. Activated macrophages produce multiple cytokines and chemokines that form key effector axes in the RA synovial inflammatory network. TNF-α and IL-6 promote cytokine cascade amplification, acute-phase responses, and immune-cell activation. IL-1β is closely associated with enhanced synovial inflammation, cartilage matrix degradation, and local tissue destruction. IL-18 can further promote inflammatory T-cell responses and interferon-γ (IFN-γ)-associated immune responses, thereby aggravating local synovial inflammation (18, 22).

Chemokine release is another major way in which macrophages contribute to persistent RA inflammation. CCL2 promotes the recruitment of peripheral blood monocytes into inflamed synovium through CCR2-related pathways, thereby providing a continuous source of inflammatory monocytes/macrophages within synovial tissue (23). CXCL8 participates in neutrophil recruitment and also acts on endothelial cells and local inflammatory regions in the synovium to promote inflammatory cell accumulation and angiogenesis (24). As monocytes, lymphocytes, and granulocytes continuously enter synovial tissue, local inflammatory cell density increases and reciprocal cellular activation intensifies, forming a positive feedback process of chemokine release, immune-cell recruitment, and further cytokine release (25). This sustained cellular recruitment and inflammatory amplification is one reason why RA synovial inflammation rarely resolves spontaneously.

Macrophage-derived inflammatory mediators also directly influence structural changes in synovial tissue. TNF-α, IL-1β, and IL-6 can act on synovial stromal cells, vascular endothelial cells, and cartilage-related cells to promote adhesion molecule expression, inflammatory cell adhesion and migration, angiogenesis, and synovial thickening (26). Under chronic inflammatory stimulation, synovial tissue gradually forms an inflammatory pannus-like structure enriched in immune cells, proliferative FLS, and newly formed vessels, thereby creating a local microenvironment permissive for cartilage invasion and bone destruction.

IL-1β and IL-18 differ from many other inflammatory cytokines because their maturation and release depend strongly on inflammasome-related pathways (27). After exposure to TLR signals, immune complexes, lipid metabolic stress, mitochondrial damage, or changes in ion flux, macrophages can promote NLRP3 inflammasome assembly and activate caspase-1, which cleaves pro-IL-1β and pro-IL-18 into mature cytokines for extracellular release (28). This process establishes a direct link among metabolic disturbance, organelle damage, and cytokine release. It also indicates that RA synovial macrophages are not passive cytokine-producing cells but key cells that convert danger signals in the synovial microenvironment into IL-1β, IL-18, and pyroptosis-related inflammatory output. Macrophage-derived inflammatory mediators, therefore, explain the cellular basis of inflammatory amplification in RA and help explain, through the NLRP3 inflammasome, how lipid metabolic abnormalities are linked to persistent synovial inflammation.

### 2.3 Crosstalk between macrophages, FLS, and osteoclast precursors

Persistent RA synovial inflammation does not result from the isolated action of one inflammatory cell type. It is generated by a pathological microenvironment jointly constructed by immune cells and synovial stromal cells. Bidirectional crosstalk between macrophages and FLS is particularly important in this process. Compared with normal synovial fibroblasts, RA-FLS show stronger proliferation, migration, resistance to apoptosis, and tissue-invasive capacity, making them a major cellular basis for synovial hyperplasia, pannus formation, and cartilage destruction (29). Macrophage-derived signals can maintain RA-FLS in a pathologically activated state, enabling the sustained expression of matrix-degrading enzymes, chemokines, and invasion-related molecules and thereby enhancing their migration and invasion toward the cartilage–synovium interface (12, 30).

RA-FLS are not passive recipients of inflammatory stimulation; they can, in turn, shape macrophage survival, recruitment, and functional states. In an inflammatory environment, RA-FLS produce granulocyte–macrophage colony-stimulating factor (GM-CSF), macrophage colony-stimulating factor (M-CSF), IL-6, and multiple chemokines, supporting the persistent retention and functional maintenance of monocytes/macrophages in the synovium. GM-CSF and M-CSF influence myeloid-cell differentiation, survival, and maintenance of inflammatory phenotypes, whereas chemokines facilitate the continuous entry of peripheral monocytes into synovial tissue and replenish inflammatory macrophage pools (31, 32). Thus, macrophages and FLS form a mutually dependent pathological cellular interaction unit: macrophages drive FLS invasion, and FLS reciprocally sustain macrophage activation and myeloid-cell accumulation in the synovium.

This cellular crosstalk is also influenced by the local synovial metabolic microenvironment. RA synovial tissue is characterized by hypoxia, lactate accumulation, glucose consumption, enhanced oxidative stress, and altered lipid mediators. These factors act on both macrophages and FLS and reshape their metabolic programs and functional outputs. Studies have shown that lactate transport-related molecules are expressed in both FLS and macrophages in RA synovium, and lactate can regulate FLS and macrophage migration, IL-6 secretion, and metabolic state, suggesting that metabolites participate in intercellular functional interactions within the synovium (33, 34). In addition, enhanced glycolysis in RA-FLS is closely associated with proliferation, migration, cytokine secretion, and invasive phenotypes, and inflammatory FLS-derived exosomes have been reported to promote M1-like macrophage polarization, glycolytic activation, and synovial inflammation (35). Macrophage-FLS crosstalk is, therefore, not only cytokine-mediated reciprocal stimulation but also shaped by metabolic pressures such as hypoxia, lactate, oxidative stress, and lipid metabolic abnormalities.

Beyond promoting synovial inflammation and cartilage invasion, macrophage-lineage cells directly contribute to RA bone erosion. Osteoclasts arise from the monocyte/macrophage lineage and mature under stimulation by signals such as M-CSF and RANKL, ultimately mediating bone resorption (20). In inflamed RA synovium, FLS, activated T cells, and stromal cells provide osteoclastogenic signals and promote the accumulation and differentiation of osteoclast precursors at the synovium–bone interface, thereby linking synovial inflammation to bone erosion. RA macrophages, therefore, act as both inflammatory executors and metabolic sensors in the synovial microenvironment. Within the RA synovium, these microenvironmental pressures reprogram macrophage lipid uptake, synthesis, oxidation, and cholesterol handling, thereby altering membrane signaling platforms, organelle homeostasis, and susceptibility to inflammasome activation. The contribution of macrophages to RA progression is closely associated with their dynamic functional states, spatial distribution, and interactions with other pathogenic cell populations rather than being determined by cell abundance alone. Across different synovial pathotypes, disease stages, and treatment conditions, macrophages cooperate with FLS, lymphocytes, endothelial cells, and osteoclast precursors to shape inflammatory persistence and tissue destruction. Therefore, macrophages are best considered as important amplifiers and regulators within the RA synovial network rather than independent drivers acting separately from other immune and stromal compartments. **Figure 1** illustrates the heterogeneity of macrophages in RA synovium and their interaction network with FLS, T/B cells, and osteoclast precursors while also indicating how microenvironmental factors such as hypoxia, lactate accumulation, and lipid metabolic abnormalities regulate macrophage functional states.

**Figure 1:**
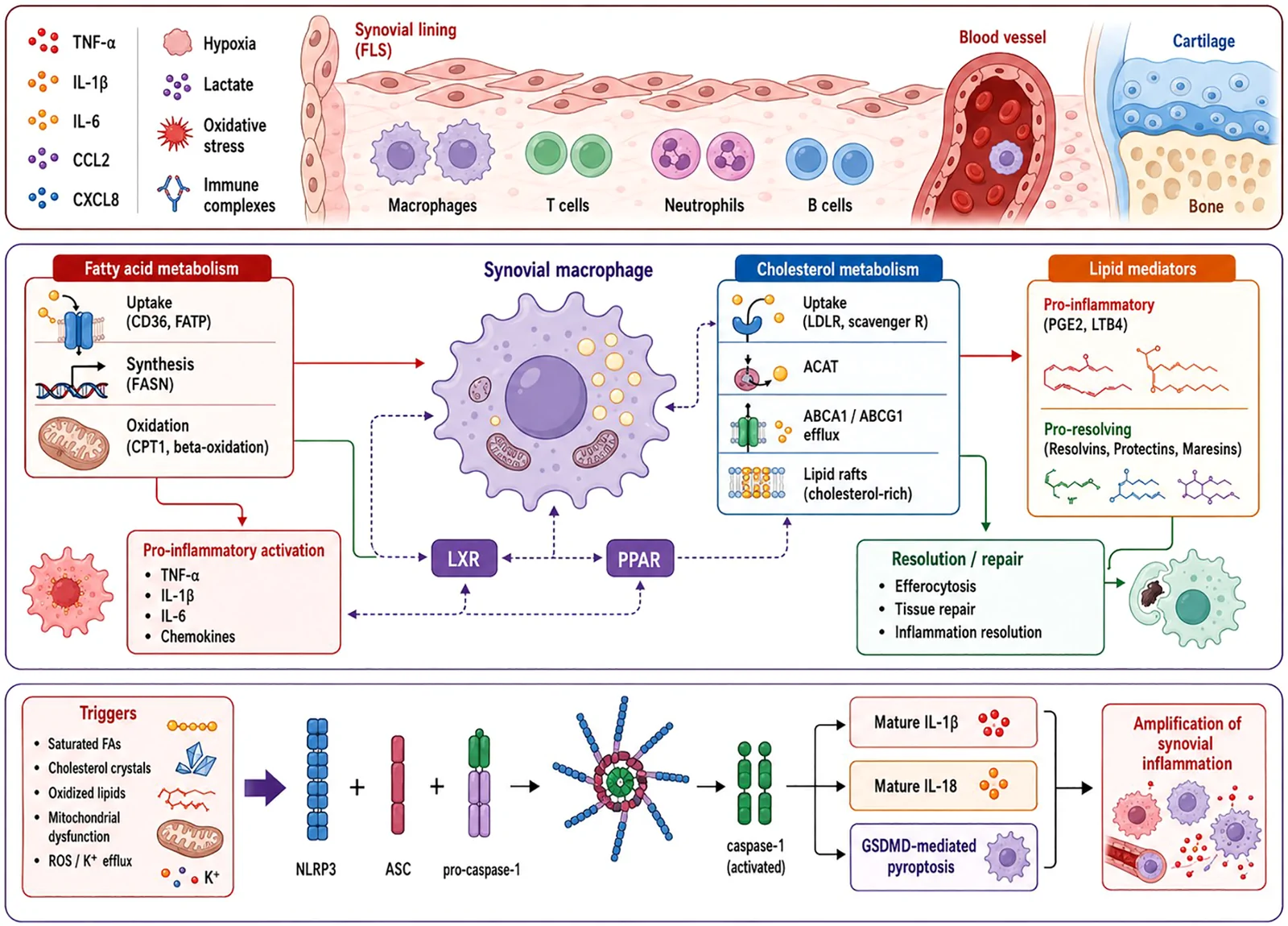
Macrophage heterogeneity and interaction networks in rheumatoid arthritis (RA) synovium. Macrophages in RA synovium display diverse origins and functional states, including tissue-resident macrophages and infiltrating inflammation-associated cells. Macrophages interact with fibroblast-like synoviocytes, T/B lymphocytes, and osteoclast precursors, contributing to inflammatory amplification, synovial hyperplasia, and bone destruction. Microenvironmental factors such as hypoxia, lactate accumulation, oxidative stress, and lipid metabolic disturbance regulate macrophage polarization and inflammasome activity. Arrows indicate key signaling interactions and directional effects.

## 3 Lipid metabolic reprogramming shapes macrophage functional states and inflammasome susceptibility

### 3.1 Metabolic features of pro-inflammatory and resolution- or repair-associated macrophage states

In RA synovium, lipid metabolic programs are better interpreted as determinants of dynamic macrophage function and inflammasome responsiveness than as fixed markers of M1/M2 polarization. The classical M1/M2 framework, nevertheless, remains useful as a metabolic reference: pro-inflammatory or M1-like macrophages stimulated by lipopolysaccharide, interferon-γ, or related stimuli are characterized by enhanced glycolysis, interruption of the tricarboxylic acid cycle, increased reactive oxygen species (ROS) production, and active synthesis of pro-inflammatory lipid mediators (36). This metabolic pattern rapidly supplies energy and biosynthetic precursors and supports inflammatory cytokine transcription, membrane renewal, and formation of inflammatory signaling platforms. At the same time, enhanced *de novo* fatty acid synthesis, active arachidonic acid metabolism, and disturbed cholesterol metabolism can further amplify pro-inflammatory signaling and maintain a high inflammatory responsiveness in macrophages (37).

By contrast, M2-like macrophages or inflammation-resolving macrophages generally rely more heavily on oxidative phosphorylation (OXPHOS), FAO, and intact mitochondrial function. Stable mitochondrial metabolism helps maintain cellular energy homeostasis and is closely related to efferocytosis, tissue repair, anti-inflammatory factor production, and inflammation resolution (38, 39). In this state, fatty acids are not simply substrates for membrane lipid synthesis or inflammatory mediator generation; they more often fuel mitochondrial oxidation and immunoregulatory programs, helping macrophages transition from inflammatory amplification toward repair and restoration of homeostasis. The direction of lipid metabolism, therefore, often determines whether macrophages remain pro-inflammatory or participate in the termination of inflammation and tissue repair.

The metabolic state of RA synovial macrophages, however, cannot be simply explained by the classic M1/M2 model. The RA synovial microenvironment is chronically hypoxic, acidic, lactate-rich, oxidatively stressed, and abnormal in lipid mediators while also being continuously stimulated by immune complexes, cytokines, chemokines, and FLS-derived signals (5). In this complex setting, synovial macrophages often display a continuous, mixed, and dynamically plastic functional spectrum. Some cells show clear pro-inflammatory transcriptional and glycolytic features, some retain phagocytic, reparative, or pro-resolving features, and others may express inflammatory and metabolic adaptation-related molecules simultaneously (18, 19). RA-specific single-cell studies further show that these macrophage states are distributed unevenly across synovial pathotypes and disease contexts. Inflammatory myeloid states characterized by IL1B, S100A12, SPP1, or related programs are enriched in active RA synovium or synovial fluid, whereas MERTK-, TREM2-, and LYVE1-associated macrophage states are more closely linked to tissue homeostasis and remission (40). These transcriptomic differences suggest subset-specific metabolic organization within RA synovium, although they do not by themselves establish fatty acid or cholesterol flux. RA synovial macrophages are thus not fixed terminal M1 or M2 states; they continuously adjust their function under combined local inflammatory and metabolic pressure. This dynamic state is particularly important from the perspective of lipid metabolism. Pro-inflammatory macrophages typically require fatty acid synthesis, membrane lipid remodeling, and pro-inflammatory lipid mediator production to support cellular activation, inflammatory signaling, and inflammasome-related responses. Inflammation-resolving macrophages, by contrast, are more closely linked to FAO, mitochondrial homeostasis, efferocytosis, and production of pro-resolving lipid mediators (41). In other words, lipid metabolism is not merely a downstream consequence of macrophage activation; it is a regulatory determinant of context-dependent functional-state transitions. In RA synovium, imbalances in lipid uptake, synthesis, oxidation, cholesterol homeostasis, and lipid mediator production may jointly shape macrophage inflammatory phenotypes and influence their roles in persistent synovial inflammation and tissue destruction. More specifically, lipid flux toward membrane synthesis and pro-inflammatory mediator production may strengthen TLR-dependent inflammasome priming, whereas impaired fatty acid oxidation and mitochondrial dysfunction may increase organelle stress and lower the threshold for NLRP3 activation.

### 3.2 Regulation of macrophage functional states by fatty acid uptake, synthesis, and oxidation

Within the RA synovial environment, fatty acid metabolism should be interpreted as a regulator of context-dependent macrophage functional programs rather than as a binary M1/M2 switch. Its inflammatory consequences depend less on substrate uptake alone than on how intracellular fatty acid flux is partitioned among membrane phospholipid synthesis, lipid-droplet storage, β-oxidation, and lipid mediator production. Macrophages promote the uptake of exogenous fatty acids through membrane-associated molecules such as CD36 and fatty acid transport proteins (FATPs), and intracellular fatty acid-binding proteins (FABPs) mediate lipid transport and distribution. Fatty acids are then activated by acyl-CoA synthetase long-chain family (ACSL) enzymes into fatty acyl-CoA and enter pathways such as membrane phospholipid synthesis, lipid droplet storage, β-oxidation, or lipid mediator generation (42, 43). Under inflammatory stimulation, enhanced fatty acid uptake and lipid redistribution provide substrates for macrophage membrane lipid remodeling, lipid droplet formation, and construction of inflammatory signaling platforms. Arachidonic acid within membrane phospholipids can also be metabolized through cyclooxygenase (COX), lipoxygenase (LOX), and cytochrome P450 pathways to generate prostaglandins, leukotrienes, and other inflammation-related lipid mediators, further contributing to inflammatory amplification (44, 45). Fatty acid uptake, therefore, influences macrophage inflammatory behavior by controlling substrate availability for membrane remodeling and lipid mediator production, whereas its effect on inflammasome susceptibility depends on the downstream metabolic fate of these lipids.

*De novo* fatty acid synthesis is more directly involved in structural remodeling and signaling maintenance during pro-inflammatory activation. TLR-related stimulation can alter macrophage lipid synthesis programs through regulatory axes such as mTORC1-SREBP1, involving key enzymes such as acetyl-CoA carboxylase (ACC) and FASN. This increases palmitate and downstream lipid products, providing a metabolic basis for membrane renewal, lipid raft formation, phagocytic responses, and inflammatory signal transduction (46, 47). Newly synthesized fatty acids can be incorporated into phospholipids, sphingolipids, and cholesterol-associated membrane structures, promote lipid raft formation and TLR4 signaling, and provide substrates for protein palmitoylation, thereby affecting membrane localization, trafficking, and complex assembly of inflammation-related proteins. Recent studies suggest that FASN-mediated fatty acid synthesis increases the availability of palmitate and palmitoyl-CoA, thereby providing lipid substrates for NLRP3 palmitoylation. This modification may facilitate NLRP3 membrane trafficking and inflammasome assembly, establishing a more direct mechanistic link between fatty acid synthesis and inflammasome activation (10, 48).

FAO has another layer of significance in determining macrophage functional outcomes. After long-chain fatty acids are activated to fatty acyl-CoA, they enter the mitochondrial matrix through the carnitine shuttle, which includes CPT1, carnitine-acylcarnitine translocase, and CPT2. β-oxidation then produces acetyl-CoA, NADH, and FADH2, linking fatty acid metabolism to the tricarboxylic acid cycle and OXPHOS and providing sustained energy (49). Compared with the rapid glycolysis and membrane lipid synthesis required for acute pro-inflammatory activation, stable FAO and mitochondrial metabolism are more common in macrophage states associated with efferocytosis, tissue repair, and inflammation resolution. During efferocytosis, in particular, macrophages must reprocess large amounts of exogenous lipids, and enhanced FAO and mitochondrial electron transport help maintain phagocytic function, anti-inflammatory factor production, and tissue repair programs (38). RA-specific evidence indicates that FAO is not uniformly associated with inflammation resolution. In circulating monocyte-derived osteoclast precursors from patients with RA, CPT1A-mediated FAO is upregulated and promotes precursor fusion and osteoclastogenesis, linking fatty acid utilization to bone erosion; however, this finding should not be interpreted as direct evidence of enhanced FAO in RA synovial macrophages (50).

The key issue in fatty acid regulation of macrophage functional states is not whether uptake, synthesis, or oxidation increases in isolation but how fatty acid metabolic flux is redistributed. In an inflammatory environment, fatty acids are more likely to be used for membrane lipid remodeling, lipid raft construction, arachidonic acid metabolism, and inflammasome assembly, thereby supporting pro-inflammatory activation. During inflammation resolution, fatty acids more often enter mitochondrial oxidation and pro-resolving lipid mediator pathways, facilitating efferocytosis, tissue repair, and restoration of immune homeostasis (38). For RA synovial macrophages, chronic inflammatory stimulation, hypoxia, oxidative stress, and abnormal lipid substrates may disrupt this metabolic flux balance, causing fatty acid metabolism to remain biased toward the maintenance of pro-inflammatory signaling and inflammatory mediator generation and thereby contributing to chronic synovial inflammation. Accordingly, fatty acid flux may influence distinct stages of inflammasome regulation: membrane lipid remodeling supports TLR-dependent priming, and FASN-derived palmitoyl-CoA may facilitate NLRP3 trafficking and assembly, whereas impaired mitochondrial fatty acid oxidation increases ROS-associated activation signals.

### 3.3 Cholesterol metabolism, membrane remodeling, and inflammasome regulation in macrophages

Cholesterol metabolism is a major regulator of macrophage membrane architecture and inflammatory signaling intensity. Unlike fatty acids, which mainly support energy supply, membrane lipid synthesis, and lipid mediator generation, cholesterol more directly affects plasma membrane fluidity, membrane microdomain stability, and receptor signal transduction (51). When free cholesterol increases in macrophage membranes, lipid rafts form more readily and remain more stable. These rafts are important platforms where Toll-like receptors, co-receptors, and downstream signaling molecules cluster (52). Under inflammatory stimulation, TLR4 and its co-receptor CD14 can accumulate in cholesterol-enriched lipid rafts, promoting receptor complex formation, MyD88-dependent signal initiation, and TLR4 endocytosis. Subsequent TRIF-dependent signaling and activation of downstream pathways such as NF-κB and MAPK can further increase macrophage responsiveness to inflammatory stimuli (53, 54). Cholesterol metabolic disturbance is, therefore, not merely lipid accumulation; it can directly amplify inflammatory signaling through membrane remodeling. By enhancing lipid-raft-dependent TLR–NF-κB signaling, membrane cholesterol enrichment may predominantly facilitate the priming phase of NLRP3 activation, increasing the expression of NLRP3 and pro-IL-1β.

Cholesterol homeostasis depends on the balance among uptake, synthesis, esterification, transport, and efflux. Macrophages take up modified lipoproteins or oxidized lipids through scavenger receptors and store cholesterol esters in lipid droplets through ACAT-mediated esterification to reduce free cholesterol toxicity. At the same time, transporters such as ABCA1 and ABCG1 promote cholesterol efflux and maintain plasma membrane cholesterol content and intracellular lipid homeostasis (55). When cholesterol efflux is impaired or cholesterol uptake is excessive, free cholesterol can become abnormally enriched in the macrophage plasma membrane and organelle-associated membranes such as the ER. This increases the stability of lipid raft-associated signaling platforms, enhances trafficking of inflammatory receptors such as TLRs to lipid rafts, and makes macrophages more likely to maintain a hyperresponsive pro-inflammatory state (56). As an oxysterol-sensing nuclear receptor, LXR promotes cholesterol efflux by regulating ABCA1, ABCG1, and related molecules and is, therefore, an important regulatory node linking cholesterol homeostasis to macrophage inflammatory responses (57).

Beyond its contribution to TLR–NF-κB-dependent priming, intracellular cholesterol overload and cholesterol crystals can promote the activation phase of the NLRP3 inflammasome by inducing lysosomal and other organelle damage. After excess cholesterol or cholesterol crystals are taken up by macrophages, they can induce phagolysosomal membrane damage, cathepsin release, and ionic homeostasis changes such as K+ efflux, thereby promoting NLRP3 inflammasome assembly and IL-1β maturation (58). Abnormal transport of cholesterol to organelle-associated membrane regions such as the ER can also induce ER stress and CaMKII/JNK/NLRP3-related inflammatory signaling. Lipid burden-induced mitochondrial dysfunction and increased ROS further act as danger signals that promote NLRP3 inflammasome activation (59). Thus, cholesterol metabolic disorder links membrane architectural changes, organelle stress, and inflammasome activation, transforming macrophages from simple inflammatory response cells into metabolic stress-sensing cells that continuously output inflammatory cytokines such as IL-1β and IL-18.

In human RA, the LXR pathway is markedly upregulated in synovial macrophages, and LXR ligands present in synovial fluid can potentiate TLR-driven cytokine release, providing direct evidence that cholesterol sensing is functionally coupled to local inflammatory activation. RA synovial fluid also contains increased oxidized lipid products and soluble LOX-1 associated with inflammatory activity, supporting a joint-specific oxidized-lipid environment (60, 61). However, direct evidence that cholesterol crystallization is a major driver of NLRP3 activation in RA synovium remains limited. If cholesterol efflux is insufficient or intracellular free cholesterol accumulates persistently, macrophage lipid rafts become more stable, TLR-related inflammatory signaling and the NLRP3 inflammasome are more easily activated, and persistent synovial inflammation is promoted (62). Taken together, cholesterol dysregulation may regulate the NLRP3 inflammasome at two interconnected levels in RA macrophages: membrane cholesterol enrichment amplifies TLR–NF-κB-dependent priming, whereas intracellular cholesterol overload and crystallization generate lysosomal, mitochondrial, and ionic stress that promotes inflammasome activation.

From a therapeutic perspective, statins provide a clinically established means of modifying cholesterol metabolism through the inhibition of 3-hydroxy-3-methylglutaryl coenzyme A reductase (HMGCR). In addition to lowering circulating cholesterol, statins may influence inflammatory signaling through the mevalonate pathway and thereby modify macrophage inflammatory responsiveness. Clinical studies in RA have provided evidence for both anti-inflammatory and cardiovascular effects. In the Trial of Atorvastatin in Rheumatoid Arthritis (TARA), adjunctive atorvastatin (40 mg/day) produced a modest but significant reduction in DAS28, C-reactive protein, and erythrocyte sedimentation rate compared with placebo (63). In the larger TRACE RA trial, atorvastatin (40 mg/day) significantly reduced LDL cholesterol and C-reactive protein and was associated with a numerically lower rate of cardiovascular events, although the primary cardiovascular endpoint did not reach statistical significance because the trial was terminated early owing to a lower-than-expected event rate (64). Thus, statins represent clinically relevant lipid-modifying agents in RA, although their role should be considered primarily in cardiovascular risk management rather than as established disease-modifying antirheumatic therapy.

### 3.4 Lipid mediators and the balance between inflammatory amplification and inflammation resolution

Lipid mediators are an important form through which macrophage lipid metabolism is converted into inflammatory functional output. Unlike fatty acid uptake, synthesis, and oxidation, which mainly reflect metabolic flux, lipid mediators directly participate in intercellular communication and rapidly alter the functional states of macrophages, endothelial cells, FLS, and infiltrating immune cells (65). Under inflammatory stimulation, arachidonic acid in membrane phospholipids is released by phospholipase A2 and further converted through cyclooxygenase, lipoxygenase, and cytochrome P450 pathways into prostaglandins, leukotrienes, thromboxanes, hydroxylated fatty acids, and other active molecules (66). These lipid mediators together constitute a dynamic regulatory network balancing inflammatory amplification and inflammation resolution in RA synovium. Direct profiling of human RA synovial fluid has identified concurrent activation of inflammatory and pro-resolving lipid mediator pathways, indicating that the joint lipid-mediator milieu is not uniformly pro-inflammatory (67). Circulating specialized pro-resolving mediator profiles also vary across RA synovial pathotypes and are associated with DMARD responsiveness, supporting disease-specific heterogeneity in inflammation-resolution capacity (68). Rather than functioning uniformly as direct NLRP3 activators, lipid mediators may shape an inflammasome-permissive synovial environment by regulating TLR–NF-κB signaling, immune-cell recruitment, oxidative stress, efferocytosis, and inflammation-resolution programs.

During inflammatory amplification, arachidonic-acid-derived lipid mediators can strengthen pro-inflammatory macrophage functions. Prostaglandin E2, leukotriene B4, and related mediators promote vasodilation, vascular permeability, pain sensitization, and inflammatory cell recruitment. By acting on macrophages and other synovial cells, they also influence the expression of TNF-α, IL-1β, IL-6, chemokines, and matrix degradation-related molecules (69). Leukotriene B4 is particularly chemotactic and promotes neutrophil and monocyte accumulation at inflammatory sites, whereas prostaglandins regulate macrophage cytokine release, vascular responses, and FLS activation through different receptor subtypes (70). Some lipid mediators are not unidirectionally pro-inflammatory in all contexts; their effects depend on receptor type, local concentration, disease stage, and cellular composition (71). Thus, in RA synovium, the more critical issue is not the elevation of a single lipid mediator but whether a broader shift in the pro-inflammatory lipid mediator profile persistently maintains macrophages and synovial cells in a hyperresponsive state.

In contrast to pro-inflammatory lipid mediators, inflammation resolution is not a passive fading of inflammatory signals but an active process governed by a series of pro-resolving lipid mediators (72). Specialized pro-resolving mediators (SPMs), including resolvins, protectins, and maresins, limit further neutrophil recruitment, promote macrophage efferocytosis of apoptotic cells and debris, reduce cytokine release, and drive macrophages toward pro-resolving and tissue-repair-associated functional states (73). In chronic synovial inflammation, the significance of SPMs is not simply anti-inflammation; rather, they promote the restoration of inflammation termination programs, enabling macrophages to shift from sustained inflammatory mediator release toward damage clearance, homeostatic rebuilding, and restriction of tissue destruction (74).

The lipid mediator network can, therefore, be viewed as a directional regulator of macrophage functional outcomes in RA. When arachidonic-acid-derived pro-inflammatory mediators remain dominant, macrophages are more likely to maintain pro-inflammatory activation, cellular recruitment, and synovial-tissue-invasion-related functions. When SPM generation is insufficient or pro-resolving signaling is impaired, the synovial microenvironment may fail to complete inflammation resolution even if cytokine levels decline to some extent (75). One major consequence of abnormal lipid metabolism in RA synovial macrophages is, therefore, disruption of the balance between pro-inflammatory and pro-resolving lipid mediators, shifting synovial inflammation from an acute defensive response to a chronic, persistent inflammatory state.

### 3.5 Lipidomics and metabolomics in biomarker discovery for RA

Lipidomics and metabolomics provide a systems-level approach for characterizing metabolic heterogeneity and identifying biomarkers of RA progression and treatment response. Untargeted profiling of serum and synovial fluid has identified lipid signatures that distinguish preclinical RA, active disease, and sustained remission and that change after antirheumatic treatment (76). In a plasma lipidomic study of 278 treated patients, fatty acids and lysophosphatidylcholines were independently associated with DAS28-ESR after adjustment for age, body mass index, and medication use (77). More recently, serum metabolomics in 220 patients with early RA from the NORD-STAR cohort identified metabolites associated with baseline disease activity and 24-week remission, although predictive performance remained moderate (78). Despite these advances, most current lipidomic and metabolomic studies analyze serum, plasma, or whole synovial fluid and, therefore, cannot determine which metabolic signatures originate specifically from synovial macrophages. Differences in disease stage, medication exposure, dietary status, sample processing, and analytical platforms also limit direct comparison among studies. Future work should integrate lipidomics and metabolomics with single-cell and spatial approaches, synovial macrophage phenotyping, and longitudinal sampling to determine whether specific metabolic signatures can support patient stratification, disease monitoring, or prediction of therapeutic response.

Macrophage lipid metabolic reprogramming, therefore, represents an interconnected regulatory network rather than a collection of isolated pathways. Membrane lipid remodeling and cholesterol enrichment mainly enhance TLR–NF-κB-dependent inflammasome priming; impaired fatty acid oxidation, lipotoxicity, oxidative stress, and organelle damage provide signals that favor NLRP3 activation; and FASN-dependent palmitate supply may support protein palmitoylation and inflammasome assembly. Meanwhile, the balance between pro-inflammatory and pro-resolving lipid mediators determines whether these responses persist or return toward tissue homeostasis. The major metabolic modules and their mechanistic relationships with macrophage inflammatory responses are summarized in **Table 2**.

**Table 2:** Major modules of macrophage lipid metabolic reprogramming and their inflammatory functions.

Metabolic module	Key molecules/pathways	Effects on macrophage function	Mechanistic position in inflammasome regulation	RA-specific evidence and relevance	References
Fatty acid uptake and intracellular transport	CD36, FATP, FABP, ACSL	Provide substrates for membrane lipid synthesis, lipid droplet formation, lipid mediator generation, and β-oxidation	Context dependent: supplies substrates for membrane remodeling, lipid mediator production, storage, or oxidation; its influence on inflammasome susceptibility depends on downstream fatty acid flux	Direct metabolic-flux evidence in RA synovial macrophages remains limited; single-cell and spatial studies nevertheless suggest subset-specific metabolic heterogeneity within RA synovium	(79)
*De novo* fatty acid synthesis	ACC, FASN, SREBP1, mTORC1	Promotes membrane lipid renewal, lipid droplet formation, phagocytic responses, and construction of inflammatory signaling platforms	Priming-related regulation: supports membrane phospholipid remodeling and lipid-raft-dependent TLR signaling, thereby favoring inflammatory transcription and inflammasome priming	Direct evidence in RA synovial macrophages remains limited; current mechanistic evidence is derived mainly from general macrophage and non-RA inflammatory models, although this pathway may provide a lipid basis for sustained inflammatory activity and inflammasome priming in RA	(80)
FASN-dependent palmitate supply and NLRP3 S-palmitoylation	FASN, palmitate, palmitoyl-CoA, ZDHHC1, ZDHHC5, ZDHHC7, ZDHHC12, ZDHHC17, ABHD17A	Provides lipid substrates for protein palmitoylation and affects membrane localization and complex assembly of signaling proteins	Trafficking and assembly: supports site-specific NLRP3 palmitoylation, Golgi-associated membrane localization, NEK7 interaction, ASC recruitment and oligomerization, and inflammasome assembly	This represents a mechanistically plausible link between lipid synthesis and inflammasome assembly, but direct validation of FASN-dependent NLRP3 palmitoylation in RA synovial macrophages is currently lacking	(81)
CPT1A-dependent FAO is increased in monocyte-derived osteoclast precursors from patients with RA and promotes osteoclast fusion, although this does not establish enhanced FAO in RA synovial macrophages	CPT1, β-oxidation, tricarboxylic acid cycle (TCA cycle), OXPHOS	Provide metabolic support for efferocytosis, IL-10 production, and tissue repair	Activation-threshold regulation: preserves mitochondrial metabolic homeostasis and limits lipotoxicity and mtROS-associated signals; impaired FAO or mitochondrial dysfunction may favor NLRP3 activation	In RA, insufficient FAO or mitochondrial dysfunction may weaken inflammation resolution	(38)
Human RA synovial fluid shows an oxidized-lipid environment and functional LXR activity, whereas direct evidence that cholesterol crystallization is a major NLRP3 trigger in RA synovium remains limited	Cholesterol accumulation, crystallization, and membrane remodeling	Enhance membrane microdomain stability and clustering of inflammatory receptors	Priming and activation: membrane cholesterol enrichment enhances lipid-raft-dependent TLR–NF-κB priming, whereas intracellular cholesterol overload or crystallization promotes lysosomal, mitochondrial, and ionic stress-related activation signals	May lower the threshold for inflammatory activation in RA synovial macrophages	(82)
Cholesterol efflux and LXR signaling	LXRα/β, ABCA1, ABCG1, APOE	Reduce free cholesterol and lipid raft cholesterol content and regulate inflammatory receptor sensitivity	Priming restraint: reduces membrane cholesterol and lipid-raft-dependent TLR–NF-κB signaling, while potentially limiting cholesterol overload-associated organelle stress; its net effect remains context dependent	An important checkpoint linking cholesterol homeostasis to inflammatory responses in RA macrophages	(83)
PPAR-mediated fatty acid sensing	PPARγ, PPARα, PPARδ	Regulates fatty acid uptake, lipid storage, FAO, and anti-inflammatory transcriptional programs	Promotes inflammatory restraint, efferocytosis, and tissue repair-related functions	May help shift RA macrophages from a pro-inflammatory state toward resolution/repair states	(84)
Arachidonic acid-derived pro-inflammatory lipid mediators	COX, LOX, CYP450, PGE2, LTB4	Promote vascular responses, pain sensitization, immune-cell recruitment, and cytokine expression	Inflammatory-context amplification: promotes leukocyte recruitment and cytokine/chemokine amplification and, for selected mediators such as LTB4, ROS-dependent inflammatory persistence; the net effect varies according to the mediator, receptor subtype, and target cell	A shift toward pro-inflammatory lipid mediators may maintain synovial hyperresponsiveness in RA	(85, 86)
Pro-resolving lipid mediators	Resolvins, protectins, maresins, SPMs	Limit neutrophil recruitment and enhance macrophage efferocytosis and tissue repair	Resolution-associated restraint: enhances efferocytosis, limits further leukocyte recruitment, and restores tissue homeostasis, thereby reducing persistent lipid, oxidative, and cellular-debris signals that maintain inflammasome susceptibility	Insufficient SPMs or impaired pro-resolving signaling may prevent termination of RA inflammation	(87)

## 4 Lipid-sensing nuclear receptor regulatory networks in RA macrophages

### 4.1 Shared and distinct regulatory features of LXR and PPAR

LXR and PPARs are ligand-activated lipid-sensing nuclear receptors that link macrophage lipid handling to inflammatory regulation, but their dominant metabolic functions differ. LXR primarily responds to intracellular oxysterol and cholesterol burden and promotes ABCA1/ABCG1-dependent cholesterol efflux, thereby regulating membrane cholesterol content, lipid-raft-associated signaling, and TLR–NF-κB-dependent inflammasome priming (88). By contrast, PPARs sense fatty acid-derived and oxidized lipid ligands and regulate isoform-specific metabolic programs: PPARγ primarily promotes fatty acid uptake and storage and supports efferocytosis and resolution-associated functions, whereas PPARα/δ more strongly enhance fatty acid oxidation and mitochondrial adaptation (89). Their functions converge on maintaining lipid homeostasis, limiting lipotoxic and organelle stress, and restraining inflammatory transcription (90). Thus, LXR and PPAR pathways should be viewed as complementary rather than interchangeable regulatory systems, while direct evidence defining their molecular crosstalk in RA synovial macrophages remains limited.

### 4.2 LXR: cholesterol homeostasis, membrane signaling, and inflammasome priming

Within the lipid metabolism–inflammasome axis, LXR functions primarily as a cholesterol-sensing checkpoint that regulates membrane cholesterol content and the intensity of TLR-dependent inflammasome priming. LXR comprises the LXRα and LXRβ isoforms and, upon sensing intracellular oxysterols, forms heterodimers with retinoid X receptor (RXR) to regulate genes involved in cholesterol transport, efflux, and lipid homeostasis (91). In macrophages, one of the core functions of LXR is to induce expression of ATP-binding cassette transporter A1 (ABCA1), ATP-binding cassette transporter G1 (ABCG1), apolipoprotein E (APOE), and related molecules. This promotes cholesterol transfer to apolipoprotein A-I or high-density lipoprotein, reduces intracellular free cholesterol burden, and maintains membrane cholesterol and intracellular lipid homeostasis (92).

For macrophages, cholesterol efflux is not only a metabolic process that prevents lipid accumulation but also a direct regulator of membrane structure and inflammatory signaling. ABCA1- and ABCG1-mediated cholesterol efflux reduces cholesterol content in the plasma membrane and lipid raft regions, limiting excessive clustering of TLRs and associated adaptor molecules in membrane microdomains and thereby modulating macrophage sensitivity to inflammatory stimuli (93). When the LXR-ABCA1/ABCG1 axis is insufficient, free cholesterol may accumulate abnormally in the plasma membrane and intracellular membrane systems. This stabilizes lipid raft-associated signaling platforms, enhances TLR-related signal transduction, and increases the risk of organelle stress and inflammasome activation (8). LXR-mediated cholesterol efflux, therefore, restrains NLRP3 signaling at two interconnected levels: it limits lipid-raft-dependent TLR–NF-κB priming and reduces intracellular cholesterol overload that can generate organelle stress-related activation signals.

LXR signaling has special significance in the RA synovial microenvironment. RA synovial macrophages are chronically exposed to cytokines, immune complexes, oxidized lipids, and cellular debris, conditions that can easily disrupt the balance among cholesterol uptake, storage, and efflux (13). If cholesterol efflux is inadequate, macrophages may develop membrane cholesterol enrichment and increased intracellular lipid-handling stress, thereby amplifying inflammatory receptor signaling and promoting the release of inflammatory mediators such as IL-1β (8). Conversely, appropriate restoration of LXR-mediated cholesterol efflux can reduce free cholesterol accumulation, improve membrane lipid composition, and limit inflammatory signal amplification induced by disrupted lipid homeostasis.

Accordingly, the LXR–ABCA1/ABCG1 axis links cholesterol handling to the inflammatory response threshold of RA synovial macrophages. The net effect of LXR signaling is context dependent because it not only controls cholesterol efflux and membrane lipid composition but also modulates NF-κB/AP-1-dependent inflammatory transcription and intersects with AMPK–mTOR–HIF-1α-regulated glycolytic reprogramming.

Beyond cholesterol efflux, LXR also regulates the transcriptional environment that determines the efficiency of NLRP3 inflammasome priming. By modulating lipid-metabolic genes and NF-κB/AP-1-dependent inflammatory transcription, LXR links cellular cholesterol status to the expression of inflammasome-related and pro-inflammatory genes (83). Upon activation, LXR regulates target genes, including ABCA1, ABCG1, and APOE, while also interfering with activation of pro-inflammatory genes by transcription factors such as NF-κB and AP-1. It thereby affects the production of macrophage cytokines, chemokines, and lipid mediators. This regulation involves both adjustment of metabolic programs and direct transcriptional repression of inflammatory genes, resulting in dual control of metabolism and inflammation.

The effects of LXR are context dependent across disease settings. Experimental studies show that in chronic inflammatory or lipid-rich microenvironments, LXR activation may slow the transcription of certain pro-inflammatory genes. However, under persistent TLR stimulation or high oxidized lipid burden, LXR-mediated metabolic changes may become coupled to inflammatory signaling, allowing macrophages to retain a degree of pro-inflammatory activation (94). LXR is, therefore, neither absolutely anti-inflammatory nor inevitably pro-inflammatory; its effect depends on the type of local stimulus, lipid load, and energy metabolic state.

In RA synovial macrophages, the impact of LXR on inflammasome susceptibility is, therefore, determined by the balance among cholesterol efflux, inflammatory transcription, and glycolytic reprogramming. Reduced membrane cholesterol may restrain lipid-raft-dependent TLR–NF-κB priming, whereas persistent TLR stimulation and enhanced glycolysis may maintain pro-inflammatory gene expression and IL-1β production (95). LXR is thus better viewed as a context-dependent regulator of the inflammasome-priming environment than as a uniformly anti-inflammatory or pro-inflammatory signal.

Pro-inflammatory activation of RA synovial macrophages depends not only on lipid metabolic abnormalities but also on enhanced glycolysis. Cytokines, immune complexes, and metabolic stress in the inflamed synovial environment can promote macrophage glucose uptake and glycolytic flux, making cells more likely to adopt a pro-inflammatory metabolic state characterized by HIF-1α, mTOR, and related glycolytic enzymes (95). Studies have shown that RA serum can induce glycolytic reprogramming in macrophages and promote IL-1β expression through the glycolysis–HIF-1α axis, suggesting that enhanced glycolysis primarily reinforces the priming environment by sustaining pro-IL-1β expression and pro-inflammatory transcription rather than acting as an independent activation signal for NLRP3 (96).

Against this background, the significance of LXR should not be limited to cholesterol metabolism. LXR signaling can intersect with energy metabolic pathways such as AMPK, mTOR, and HIF-1α, thereby influencing macrophage glycolysis and inflammatory phenotype (97). RA-related experimental studies have shown that the LXR inverse agonist SR9243 alleviates adjuvant-induced arthritis and protects against bone erosion. This effect is associated with the regulation of macrophage glycolytic metabolism, inhibition of M1-like polarization, and suppression of inflammatory activation. These findings further suggest that the AMPK/mTOR/HIF-1α pathway may mediate the influence of LXR on glycolysis and inflammatory responses.

These findings indicate that LXR bridges lipid metabolism, energy metabolism, and inflammatory activation in RA macrophages (95). On one hand, LXR-related pathways influence membrane signaling responses through cholesterol efflux and lipid homeostatic regulation. On the other hand, changes in LXR activity may alter macrophage function by regulating glycolytic flux, HIF-1α stability, and pro-inflammatory cytokine expression (8). LXR is, therefore, not merely a nuclear receptor controlling lipid storage or cholesterol transport; it is a metabolic checkpoint that integrates lipid load, energy metabolism, and inflammatory transcriptional programs (95). Importantly, LXR activity in RA is strongly context dependent. Previous studies found that LXR pathways are markedly upregulated in RA synovial macrophages and that LXR ligand activation can enhance TLR-driven cytokine release under certain conditions. In contrast, inverse modulation of LXR with agents such as SR9243 can attenuate experimental arthritis by suppressing glycolysis and M1-like activation (98). Thus, LXR cannot be simply defined as either anti-inflammatory or pro-inflammatory; its final effect depends on local synovial lipid load, TLR stimulation intensity, glycolytic state, and mode of intervention.

Overall, LXR primarily regulates the upstream inflammasome-priming environment in RA macrophages by coordinating cholesterol efflux, lipid-raft-dependent signaling, and glycolytic transcription. Its effects are strongly influenced by synovial lipid load, TLR stimulation, and cellular metabolic state, which may explain why LXR activation and inverse modulation produce different inflammatory outcomes under different experimental conditions.

### 4.3 PPARs: fatty acid handling, mitochondrial adaptation, and inflammation resolution

#### 4.3.1 PPARγ and resolution-associated macrophage programs

Within the lipid metabolism–inflammasome axis, PPARγ functions primarily as a fatty-acid-sensing regulator of lipid handling, mitochondrial homeostasis, and inflammatory transcription. As a ligand-activated transcription factor, PPARγ forms heterodimers with RXR, recognizes endogenous lipid signals such as fatty acid derivatives, oxidized lipids, and prostaglandin metabolites, and regulates genes involved in lipid uptake, storage, utilization, and inflammatory responses (99). In contrast to the cholesterol-centered role of LXR in membrane signaling and inflammasome priming, PPARγ may reduce the lipotoxic and organelle-stress conditions that favor NLRP3 activation while promoting efferocytosis and resolution-associated macrophage functions (100).

At the metabolic level, PPARγ induces the expression of CD36, lipoprotein lipase, fatty acid-binding proteins, lipid-droplet-associated proteins, and related molecules, thereby promoting macrophage lipid uptake, transport, and storage. Moderate lipid storage helps convert excess free fatty acids into relatively stable lipid droplets, reducing lipotoxicity and organelle stress. PPARγ is also linked to FAO, mitochondrial function, and efferocytosis, providing metabolic support for inflammation-resolving macrophages (101). During efferocytosis, macrophages must handle a substantial lipid load derived from cell membranes, and PPARγ-mediated lipid-handling programs help maintain phagocytic function and anti-inflammatory responses, thereby promoting tissue homeostasis. At the level of inflammatory transcriptional regulation, PPARγ activation generally suppresses pro-inflammatory transcriptional networks such as NF-κB, AP-1, and STAT, reducing the expression of TNF-α, IL-1β, IL-6, CCL2, and related inflammatory mediators (101). Its anti-inflammatory effects are not limited to the competitive inhibition of cytokine transcription; they also involved stabilization of transcriptional co-repressor complexes, reduced inflammatory gene promoter activity, and decreased sensitivity of pro-inflammatory signaling pathways (101). Meanwhile, PPARγ promotes the expression of IL-10, MRC1, MERTK, and other molecules associated with immune regulation, efferocytosis, and tissue repair, shifting macrophages from sustained pro-inflammatory states toward functions characterized by resolution and repair (102).

In the RA synovial environment, PPARγ may interrupt the cycle of lipid load, inflammatory activation, and tissue injury. RA synovial macrophages are chronically exposed to cytokines, hypoxia, oxidized lipids, and metabolic stress, conditions that readily maintain pro-inflammatory and hyperresponsive states (101). PPARγ activation can improve fatty acid handling and lipid storage, reducing cellular stress induced by free lipids, and can suppress pro-inflammatory transcriptional programs, promoting macrophage transition toward inflammatory restraint, efferocytosis, and tissue repair (101). RA-specific evidence further shows altered PPARG expression in patient synovial tissue and peripheral blood mononuclear cells, while mechanistic experiments in macrophages and collagen-induced arthritis models indicate that PPARG-driven autophagy enhances ROS clearance and suppresses macrophage inflammatory polarization and NLRP3 inflammasome activation (99).

The influence of PPARγ on inflammasome signaling can be understood at both the priming and activation levels. By suppressing NF-κB- and AP-1-dependent inflammatory transcription, PPARγ may limit the expression of NLRP3 and pro-IL-1β and thereby restrain inflammasome priming (103, 104). By promoting lipid storage, fatty acid utilization, mitochondrial homeostasis, and efferocytosis, PPARγ may also reduce lipotoxicity, mtROS accumulation, and organelle-stress signals that favor NLRP3 activation (105). These effects remain dependent on ligand identity, inflammatory stimulus intensity, macrophage functional state, and crosstalk with RA-FLS. PPARγ should, therefore, be viewed as a context-dependent regulator of macrophage metabolic adaptation and inflammasome susceptibility rather than as a marker of a fixed M2-like phenotype.

#### 4.3.2 PPARα/δ, FAO, and immunometabolic regulation

In addition to PPARγ, PPARα and PPARδ are important nuclear receptors through which macrophages sense fatty acid status and regulate immunometabolism. Both respond to fatty acids and their derivatives and regulate the expression of genes involved in fatty acid uptake, mitochondrial β-oxidation, peroxisomal lipid metabolism, and energy homeostasis (106). Whereas PPARγ is more closely linked to lipid storage, efferocytosis, and anti-inflammatory transcriptional programs, PPARα and PPARδ are more prominently involved in fatty acid catabolism and oxidative metabolic regulation, thereby affecting macrophage functional outcomes under inflammatory, reparative, and metabolic stress conditions.

PPARα mainly participates in fatty acid catabolism and lipid load handling. Its activation upregulates FAO-related molecules such as CPT1, ACOX1, and MCAD, promotes the metabolism of long-chain fatty acids in mitochondria or peroxisomes, and reduces the accumulation of free fatty acids and lipotoxic intermediates. For macrophages chronically exposed to lipid burden and oxidative stress, PPARα-mediated fatty acid utilization helps reduce organelle stress induced by lipid overload and limits the sustained amplification of pro-inflammatory signals (107). PPARα can also interfere with inflammatory transcriptional pathways such as NF-κB and AP-1, reducing the expression of some pro-inflammatory factors and linking fatty acid metabolic regulation to inflammatory suppression.

PPARδ is closely related to oxidative metabolism, mitochondrial function, and tissue-repair-associated programs in macrophages. PPARδ activation promotes the expression of genes involved in FAO and OXPHOS, increases macrophage capacity to use lipid substrates, and supports energetic demands during tissue repair, efferocytosis, and inflammation resolution (49). In some inflammatory models, PPARδ signaling is thought to drive macrophages toward immunoregulatory or repair-associated states, reduce excessive cytokine release, and improve metabolic adaptation after local tissue injury (108). This effect does not simply fix macrophages in an M2-like state. Rather, by enhancing lipid oxidation and mitochondrial homeostasis, it may help macrophages exit sustained pro-inflammatory responses.

PPARα/δ-related mechanisms may be relevant in RA synovium. RA synovial macrophages are chronically exposed to cytokines, hypoxia, oxidative stress, and abnormal lipid metabolism. Impaired fatty acid utilization may lead to the accumulation of lipid intermediates, mitochondrial dysfunction, and enhanced inflammatory signaling (109). By promoting FAO, maintaining mitochondrial function, and improving lipid substrate handling, PPARα and PPARδ may theoretically reduce lipotoxic stress and influence macrophage cytokine release, phagocytic capacity, and efficiency of inflammation resolution (50). They may, therefore, represent important molecular nodes regulating metabolic adaptation and inflammatory intensity in RA macrophages.

The effects of PPARα/δ on inflammasome susceptibility are context dependent. By promoting mitochondrial and peroxisomal fatty acid oxidation, these receptors may limit the accumulation of lipotoxic intermediates, preserve mitochondrial metabolic homeostasis, and reduce stress signals that favor NLRP3 activation (105, 110). Enhanced oxidative metabolism, however, is not uniformly anti-inflammatory. When mitochondrial integrity is impaired or lipid influx exceeds oxidative capacity, increased fatty acid utilization may fail to restore metabolic homeostasis and may even sustain oxidative stress (111). Together with PPARγ, PPARα/δ, therefore, complement the cholesterol-centered actions of LXR by regulating the fatty acid–mitochondrial arm of inflammasome control rather than the lipid raft–TLR priming axis.

### 4.4 Integrated regulation by LXR and PPAR: functional convergence, context dependence, and therapeutic implications

Although LXR and PPAR regulate distinct branches of macrophage lipid metabolism, their downstream effects converge on cellular lipid homeostasis, inflammatory transcription, organelle stress, and inflammation-resolution capacity. Direct molecular crosstalk between these nuclear receptors in RA synovial macrophages remains incompletely defined; therefore, their therapeutic relevance is better considered within a shared, context-dependent regulatory framework rather than as uniformly anti-inflammatory pathways. Preclinical studies indicate that pharmacological modulation of both LXR and PPAR pathways can influence arthritis-related inflammation, although through distinct metabolic mechanisms. LXR-directed interventions mainly affect cholesterol efflux, membrane signaling, and glycolysis-associated inflammatory priming, whereas PPAR-directed interventions primarily regulate fatty acid handling, mitochondrial metabolism, efferocytosis, and resolution-associated functions (83, 95). These findings support the consideration of LXR and PPAR within a shared therapeutic framework while indicating that treatment outcomes may vary according to receptor subtype, mode of intervention, cellular target, and metabolic context.

However, therapeutic modulation of LXR or PPAR entails important translational limitations. Both receptor families are broadly expressed beyond macrophages, including in the liver, adipose tissue, vascular endothelium, FLS, and other immune cells (112). Systemic modulation may, therefore, alter cholesterol and fatty acid metabolism, glucose homeostasis, insulin sensitivity, body weight, fluid balance, and cardiovascular risk. These concerns are particularly relevant in RA, in which chronic inflammation frequently coexists with metabolic abnormalities and increased cardiovascular risk. Receptor-targeted strategies, therefore, require careful evaluation of tissue selectivity, dose, and systemic metabolic effects.

Furthermore, the consequences of LXR and PPAR modulation are determined by receptor subtype, cellular target, and metabolic context. LXR activity may produce divergent inflammatory outcomes according to synovial lipid load, TLR stimulation, and glycolytic state, whereas PPAR isoforms differ in their effects on lipid storage, fatty acid oxidation, and resolution-associated programs. Accordingly, activation of either pathway should not be regarded as uniformly beneficial, and therapeutic responses are likely to vary with disease stage and mode of intervention.

RA synovial inflammation is maintained by multiple factors, including autoantibodies, lymphocyte responses, macrophage activation, FLS invasion, osteoclast differentiation, and angiogenesis. Modulation of either LXR or PPAR alone is, therefore, unlikely to fully interrupt this multicellular inflammatory network. **Figure 2** illustrates the major pathways of lipid metabolic reprogramming in RA synovial macrophages and their regulatory relationships with pro-inflammatory and anti-inflammatory phenotypes.

**Figure 2:**
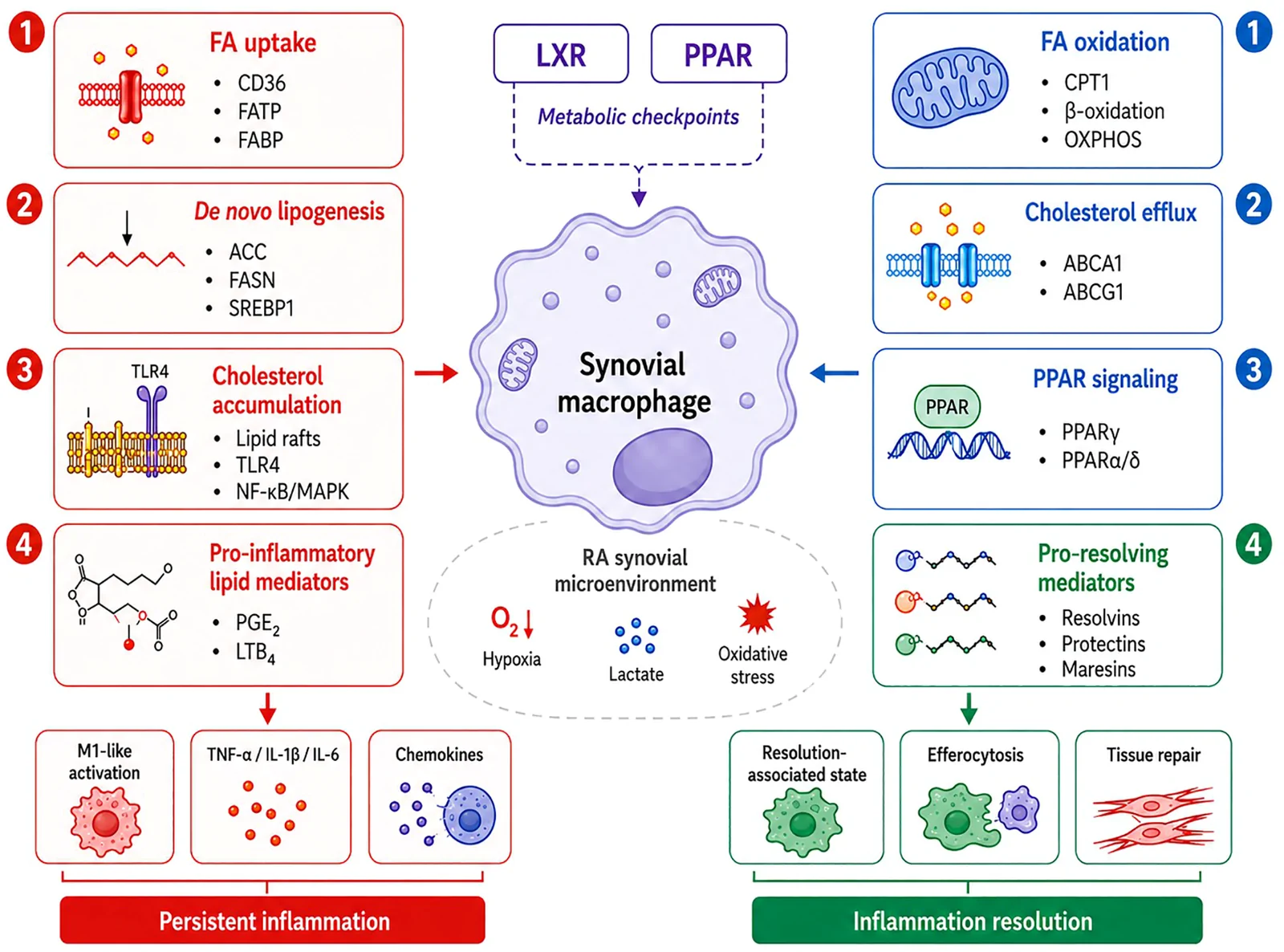
Lipid metabolic reprogramming and functional polarization of rheumatoid arthritis (RA) synovial macrophages. In RA synovium, macrophages undergo metabolic changes such as enhanced fatty acid uptake, *de novo* fatty acid synthesis, and cholesterol accumulation. Fatty acid oxidation and cholesterol efflux contribute to resolution- or repair-associated phenotypes. Liver X receptor and peroxisome proliferator-activated receptor regulate cholesterol homeostasis and fatty acid metabolism, respectively, thereby influencing pro-inflammatory and pro-resolving macrophage polarization. Arrows indicate the directional effects of metabolic pathways and signaling; the left side represents pro-inflammatory pathways, whereas the right side represents resolution- or repair-associated pathways.

## 5 Roles of *de novo* fatty acid synthesis and lipid modification in inflammasome activation

### 5.1 FASN as a metabolic enzyme supporting inflammatory activation

FASN is the key rate-limiting enzyme in *de novo* fatty acid synthesis. It converts acetyl-CoA and malonyl-CoA into saturated long-chain fatty acids such as palmitate through sequential condensation, reduction, and dehydration reactions (113). During macrophage inflammatory activation, particularly under TLR stimulation, cytokine exposure, or metabolic stress, FASN expression and activity are markedly upregulated. This provides sustained lipid substrate support to meet the energetic and structural demands of inflammatory responses (114).

FASN-mediated fatty acid synthesis not only supports macrophage energy metabolism but also provides material for membrane phospholipid renewal, ER and Golgi membrane expansion, and formation of membrane microdomains and lipid rafts. In the pro-inflammatory activation state, macrophages must rapidly assemble signaling complexes such as TLR signaling platforms and inflammasome complexes (10). Palmitate generated by FASN can be incorporated into membrane phospholipids, enhance membrane fluidity and microdomain stability, provide necessary substrates for protein membrane localization and lipid modification, and participate in the spatial organization of membrane-associated signaling complexes.

Mechanistically, FASN may connect *de novo* fatty acid synthesis to inflammasome regulation through two related routes. Newly synthesized fatty acids contribute to membrane phospholipid renewal and organelle remodeling, thereby influencing lipid raft organization and the membrane platforms required for inflammatory signaling (46). More specifically, FASN-derived palmitate can be converted to palmitoyl-CoA, which serves as the acyl donor for protein S-palmitoylation and may facilitate NLRP3 subcellular trafficking and inflammasome assembly (10). FASN may, therefore, affect both the signaling environment that supports inflammasome priming and the lipid-modification-dependent processes that organize NLRP3 assembly (115). Direct evidence for this dual mechanism in RA synovial macrophages remains limited, and its relevance should be distinguished from findings obtained in general macrophage or non-RA inflammatory models.

### 5.2 Lipid synthesis, organelle remodeling, and inflammasome assembly

NLRP3 inflammasome activation is not induced by a single inflammatory signal alone; it depends on subcellular localization, organelle communication, and membrane structural changes (28). At rest, NLRP3 remains at a relatively low activation level. After macrophages are stimulated by ATP, crystal particles, lipid stress, mitochondrial damage, lysosomal rupture, or related triggers, NLRP3 undergoes conformational change, subcellular translocation, NEK7 binding, ASC recruitment, and caspase-1 activation, eventually forming a multiprotein complex with inflammatory proteolytic activity (116). This process is highly dependent on the states of organelles such as the ER, Golgi apparatus, mitochondria, and lysosomes.

The ER is a major site of lipid synthesis and membrane protein processing and also serves as an important membrane scaffold during inflammasome activation. Inflammatory stimulation can alter ER membrane lipid composition, membrane tension, and calcium homeostasis, inducing ER stress and the unfolded protein response (117). When *de novo* fatty acid synthesis is enhanced, newly synthesized fatty acids can enter phospholipids, sphingolipids, and cholesterol-related membrane structures, influencing ER membrane fluidity, curvature, and contacts with other organelles. If lipid synthesis becomes imbalanced, the ER may shift from a lipid metabolic site to a platform for inflammatory signal amplification, creating favorable conditions for NLRP3 activation. The Golgi apparatus and associated membrane structures also participate in NLRP3 assembly (27). Studies suggest that during NLRP3 activation, NLRP3 can translocate to the trans-Golgi network or dispersed Golgi-related structures. These membrane regions are enriched in specific phospholipids and negatively charged lipids, providing a spatial basis for NLRP3 recruitment and oligomerization (118). Abnormal lipid synthesis may change Golgi membrane composition, phospholipid distribution, and membrane curvature, thereby affecting interactions between NLRP3 and downstream molecules such as NEK7 and ASC. Mitochondria are both metabolic regulatory centers and sources of danger signals during NLRP3 activation (48). Abnormal lipid synthesis or increased lipid load can disrupt mitochondrial membrane integrity and OXPHOS efficiency, induce mtROS elevation, reduce membrane potential, or trigger mitochondrial DNA (mtDNA) release, thereby promoting NLRP3 inflammasome activation (119). In addition, ER–mitochondria contact sites are important interfaces for lipid transport and calcium exchange. Abnormal lipid composition may disturb ER–mitochondrial communication and further aggravate organelle stress. Lysosomes mainly participate in particle- or crystal-induced NLRP3 activation (120). After macrophages engulf cholesterol crystals, urate crystals, or other poorly degradable particles, lysosomal membrane damage, cathepsin release, and ionic homeostasis disruption can trigger NLRP3 assembly. Lipid metabolic abnormalities may also increase lysosomal burden through cholesterol accumulation, altered membrane lipid composition, or impaired autophagy-lysosome function, making lysosomes more likely to become upstream triggers of inflammasome activation (28).

Taken together, organelle stress and membrane remodeling represent mechanistically distinct but interconnected components of the lipid metabolism–inflammasome axis. Lysosomal and mitochondrial injury primarily provide activation-associated danger signals, whereas remodeling of Golgi-associated membranes establishes a spatial platform for NLRP3 recruitment and oligomerization. FASN-dependent palmitate production further couples *de novo* lipogenesis to protein S-palmitoylation, thereby potentially regulating NLRP3 membrane trafficking and inflammasome assembly. Collectively, these processes define a mechanistic framework through which dysregulated lipid synthesis may be translated into IL-1β/IL-18 maturation and pyroptosis-associated inflammatory amplification in RA synovium. However, the specific contribution of the FASN–palmitoylation axis remains to be directly validated in RA synovial macrophages.

### 5.3 Protein palmitoylation: a bridge linking lipid metabolism to NLRP3 activation

Protein palmitoylation is an important reversible lipid modification in which palmitate is typically attached to cysteine residues through thioester bonds, thereby altering protein hydrophobicity, membrane affinity, subcellular localization, and protein–protein interactions. Compared with phosphorylation or ubiquitination, palmitoylation more directly connects lipid metabolic status with the spatial localization of signaling proteins. NLRP3 inflammasome activation is not merely a consequence of increased molecular expression; it also requires NLRP3 recruitment, trafficking, and complex assembly on specific intracellular membrane structures. Protein palmitoylation, therefore, provides a more specific mechanistic entry point for understanding how lipid synthesis regulates inflammasome activation.

#### 5.3.1 FASN-dependent palmitate supply

*De novo* fatty acid synthesis mediated by FASN mainly generates palmitate, which can be activated to palmitoyl-CoA and serve as the lipid substrate for protein palmitoylation. Thus, FASN does not merely provide fatty acid raw materials for membrane synthesis; it may also regulate the localization and function of inflammation-related proteins by influencing palmitoylation substrate supply (10). During macrophage inflammatory activation, enhanced fatty acid synthesis can increase palmitate and palmitoyl-CoA levels, providing a metabolic basis for palmitoylation of multiple signaling proteins.

Protein palmitoylation is usually mediated by the DHHC domain-containing palmitoyltransferase family and can be reversed by depalmitoylating enzymes, making it dynamically regulated (48). This reversibility allows cells to rapidly adjust the distribution of proteins on the ER, Golgi apparatus, plasma membrane, or other membranous organelles according to inflammatory stimuli and metabolic state (121). In inflammatory signaling pathways, palmitoylation can bring signaling molecules closer to specific membrane platforms, enhance their interactions with adaptor proteins or downstream effectors, and thereby increase the efficiency of signaling complex assembly.

FASN-dependent palmitate supply can, therefore, be viewed as an important prerequisite for lipid synthesis to participate in inflammatory signal regulation. When FASN activity increases, macrophages obtain not only more raw materials for membrane lipid synthesis but also greater capacity for protein lipid modification. When FASN is inhibited, reduced palmitate supply may weaken membrane localization and the complex assembly of certain inflammatory signaling proteins. This mechanism provides a metabolic basis linking FASN to the NLRP3 inflammasome.

#### 5.3.2 Site-specific NLRP3 S-palmitoylation: ZDHHC palmitoyltransferases, membrane trafficking, and inflammasome assembly

Current evidence indicates that NLRP3 S-palmitoylation is site- and enzyme-specific rather than a uniform membrane-anchoring event. ZDHHC7-mediated palmitoylation of NLRP3 at Cys126 promotes its localization to the trans-Golgi network and dispersed trans-Golgi network, thereby facilitating ASC recruitment and oligomerization. ZDHHC1-mediated palmitoylation at Cys130 and Cys958 further regulates NLRP3 trafficking among the Golgi apparatus, mitochondria, endosomes, and the microtubule-organizing center (121, 122). In addition, FASN-dependent palmitoylation at Cys898 promotes NLRP3 translocation to dispersed Golgi membranes, although the specific ZDHHC enzyme responsible for this site remains unclear (10).

At the level of inflammasome oligomerization and assembly, ZDHHC5-mediated palmitoylation of the NLRP3 leucine-rich repeat domain promotes NLRP3 oligomerization, NLRP3–NEK7 interaction, and the formation of large intracellular ASC aggregates (48). ZDHHC17-mediated palmitoylation of NLRP3 at Cys419 likewise enhances NLRP3–NEK7 binding and promotes inflammasome activation (123). These findings together indicate that NLRP3 S-palmitoylation regulates sequential processes extending from membrane trafficking to NEK7 engagement, ASC oligomerization, and inflammasome assembly.

The significance of this finding is that it elevates lipid metabolism from a background metabolic state to a direct regulatory factor in inflammasome assembly. Previous studies of NLRP3 activation have emphasized danger signals such as K+ efflux, ROS, lysosomal damage, and mitochondrial stress. The mechanism of NLRP3 palmitoylation indicates that efficient inflammasome assembly also depends on the status of lipid synthesis and protein lipid modification. In other words, FASN-mediated fatty acid synthesis can influence where NLRP3 goes inside the cell and with which molecules it assembles by providing substrates for palmitoylation, thereby regulating the intensity of inflammasome activation.

#### 5.3.3 Depalmitoylation and negative regulation of NLRP3 signaling

Because S-palmitoylation is reversible, NLRP3 activity is also controlled by depalmitoylation and degradation mechanisms. ABHD17A removes ZDHHC5-dependent palmitoylation from NLRP3, thereby limiting NLRP3–NEK7 interaction and inflammasome activation. Notably, palmitoylation does not invariably promote NLRP3 activation. ZDHHC12-mediated NLRP3 palmitoylation facilitates its recognition by the HSC70–LAMP2A machinery and promotes chaperone-mediated autophagic degradation, thereby preventing sustained inflammasome signaling (48, 124). These findings indicate that the functional consequences of NLRP3 palmitoylation depend on the modified site, the responsible enzyme, and the stage of inflammasome activation.

#### 5.3.4 Potential significance for inflammatory responses in RA macrophages

The potential relevance of the FASN–NLRP3 palmitoylation axis to RA lies in its capacity to couple lipid synthetic activity with the spatial organization of inflammasome assembly. RA-specific studies provide indirect support for the upstream and downstream components of this framework. The rheumatoid synovial environment has been shown to alter fatty acid metabolism in human monocytes, while recent patient-based and experimental studies have linked macrophage metabolic reprogramming to NLRP3 inflammasome activation in RA (125, 126). However, these findings do not directly establish FASN-dependent NLRP3 palmitoylation in RA. Persistent inflammatory signaling, hypoxia, oxidized lipid exposure, and cellular-debris clearance impose sustained metabolic and lipid-handling pressure on RA synovial macrophages (10, 121). Under these conditions, increased FASN activity could expand the intracellular supply of palmitate and palmitoyl-CoA available for protein S-palmitoylation. Evidence from non-RA macrophage models indicates that FASN-dependent lipid synthesis and ZDHHC-mediated NLRP3 palmitoylation regulate membrane association, subcellular trafficking, NEK7 engagement, ASC recruitment, and inflammasome activation (121, 122). These findings provide a plausible mechanistic basis for proposing that dysregulated lipogenesis may amplify IL-1β/IL-18 maturation and pyroptosis-associated inflammatory output in RA synovium. However, whether ZDHHC1, ZDHHC5, ZDHHC7, ZDHHC12, ZDHHC17, ABHD17A, and the corresponding NLRP3 palmitoylation sites are dysregulated in RA synovial macrophages remains unknown and requires direct validation in primary RA synovial macrophages, synovial tissue, and experimental arthritis models.

## 6 The NLRP3 inflammasome as an integrative framework linking lipid metabolic stress to inflammatory output in RA

The effects of lipid metabolic disturbance on RA synovial macrophages must ultimately be translated into biologically active inflammatory outputs that sustain synovial inflammation and tissue destruction. The NLRP3 inflammasome is a key platform in this process. Unlike cytokines such as TNF-α and IL-6, which can be released through conventional secretory pathways, IL-1β and IL-18 require inflammasome-mediated cleavage and maturation to acquire full biological activity (127). The NLRP3 inflammasome can, therefore, be viewed as an important converter connecting lipid metabolic stress, organelle damage, and mature inflammatory cytokine release. Within this framework, cholesterol-rich lipid rafts and sustained TLR–NF-κB signaling predominantly reinforce inflammasome priming, whereas cholesterol crystals, lipotoxicity, organelle damage, ionic flux disturbances, mtROS, and lipid peroxidation provide activation-associated signals. FASN-dependent palmitate-derived acyl donors and ZDHHC-mediated NLRP3 palmitoylation may further facilitate NLRP3 membrane trafficking, NEK7 interaction, ASC recruitment, and inflammasome assembly.

### 6.1 Priming, activation, and assembly of the NLRP3 inflammasome

The NLRP3 inflammasome is mainly composed of the sensor NLRP3, the adaptor protein ASC, and the effector protein pro-caspase-1. Canonical activation typically includes priming and activation stages. The priming stage is usually induced by TLR signals, cytokines, immune complexes, or related stimuli, which activate transcription factors such as NF-κB and upregulate NLRP3, pro-IL-1β, and pro-IL-18, providing the molecular basis for subsequent inflammasome assembly (128). The activation stage is triggered by ATP, K+ efflux, crystalline particles, mitochondrial damage, lysosomal rupture, lipid metabolic stress, and related signals. These stimuli promote NLRP3 conformational change and recruitment of ASC and pro-caspase-1, forming an inflammasome complex with proteolytic activity.

In RA, ACPAs provide a disease-specific upstream signal that promotes NLRP3 inflammasome priming through inflammatory transcription and facilitates its activation through Panx1-dependent ATP release and TWIK2-mediated K+ efflux (116). This autoimmune stimulus may cooperate with lipid-derived organelle stress to sustain inflammasome responsiveness in the synovial niche. Activated caspase-1 cleaves pro-IL-1β and pro-IL-18 into their mature forms and further cleaves gasdermin D (GSDMD). The N-terminal fragment of GSDMD forms pores in the plasma membrane, enabling the extracellular release of mature IL-1β and IL-18 while inducing pyroptosis. Pyroptosis differs from simple cell death because it involves membrane disruption, release of intracellular contents, and diffusion of local danger signals and, therefore, has a marked inflammatory amplification effect (129). In RA synovial macrophages, NLRP3 activation means not only maturation and release of IL-1β and IL-18 but also further enhancement of local synovial inflammation through pyroptotic macrophage death.

In addition to the canonical pathway, non-canonical inflammasome pathways can participate in NLRP3 activation. In human cells, cytosolic lipopolysaccharide activates caspase-4/5, whereas in mouse cells it mainly activates caspase-11. These non-canonical caspases induce GSDMD cleavage and plasma membrane pore formation and indirectly promote NLRP3 inflammasome activation through mechanisms such as K+ efflux (130). Although the specific role of the non-canonical pathway in RA remains to be clarified, it indicates that inflammasome activation is not a single linear process but an effector inflammatory network generated by the convergence of multiple danger signals. Accordingly, lipid metabolic abnormalities may regulate NLRP3 through three interconnected mechanistic layers. Membrane lipid remodeling and inflammatory transcription primarily shape the priming phase; ionic flux disturbances, mitochondrial injury, lysosomal rupture, ER stress, and oxidative stress provide activation-associated signals; and post-translational modifications, particularly S-palmitoylation, regulate NLRP3 trafficking, NEK7 engagement, ASC oligomerization, and inflammasome assembly. This three-layer framework provides the organizing logic for the following sections.

### 6.2 Lipid metabolic stress signals driving the activation phase of NLRP3

#### 6.2.1 Cholesterol accumulation and lysosomal damage

Abnormal cholesterol metabolism is an important source of lipid stress-induced NLRP3 inflammasome activation. When cholesterol efflux is inadequate, modified lipid uptake increases, or cholesterol crystals form, macrophages are prone to increased intracellular cholesterol burden (131). Engulfed cholesterol crystals are difficult to degrade completely and can cause phagolysosomal membrane damage, cathepsin leakage, and ionic homeostasis changes such as K+ efflux, thereby providing activating signals for NLRP3 inflammasome assembly (132).

In RA synovium, cholesterol crystals are not a RA-specific pathological hallmark, but oxidative stress, lipid-handling pressure, and persistent inflammatory cell infiltration may together increase the risk of disturbed macrophage lipid homeostasis (133). Accordingly, the significance of cholesterol accumulation for NLRP3 is not limited to abnormal lipid storage. Through lysosomal damage and organelle stress, cholesterol accumulation can lower the activation threshold of the inflammasome and make macrophages more likely to mount strong inflammatory responses to local synovial danger signals.

#### 6.2.2 Saturated fatty acids, lipotoxicity, and mitochondrial ROS

Saturated fatty acids and lipotoxic stress are also important upstream factors for NLRP3 inflammasome activation. Excess saturated fatty acids can disrupt membrane lipid composition and induce ER stress, mitochondrial dysfunction, and abnormal OXPHOS. When mitochondria are damaged, increased mtROS, loss of membrane potential, and release of mtDNA can all serve as danger signals that promote NLRP3 activation (134). Unlike simple cytokine stimulation, lipotoxic stress can directly convert nutrient metabolic abnormalities and organelle injury into inflammasome responses.

In RA synovial macrophages, lipotoxicity does not necessarily appear as typical lipid deposition seen in adipose tissue. It is more often reflected by imbalance among fatty acid utilization, storage, and oxidation. If macrophages cannot efficiently handle excess fatty acids, lipid intermediate accumulation and mitochondrial injury can jointly enhance ROS generation, placing NLRP3 in a more readily activated state (135). Saturated fatty acid-related stress, therefore, provides an important mechanistic explanation for the link among metabolic pressure, mitochondrial damage, and inflammasome activation in RA.

#### 6.2.3 Lipid peroxidation, ferroptosis, and oxidative inflammatory amplification

Lipid peroxidation links oxidative stress to NLRP3 inflammasome activation. ROS-driven oxidation of polyunsaturated fatty acids generates reactive lipid products that disrupt plasma and organelle membranes, aggravate mitochondrial dysfunction, and further increase ROS production (136, 137). These changes destabilize ionic and organelle homeostasis and reinforce inflammasome-activating signals, creating a positive feedback loop between lipid damage and inflammatory cytokine release (138). In chronically inflamed RA synovium, this process may maintain macrophage inflammasome hyperresponsiveness.

Importantly, lipid peroxidation is also a defining biochemical feature of ferroptosis, an iron-dependent form of regulated cell death that has increasingly been implicated in RA. Human and experimental evidence indicates that RA may exhibit a paradoxical pattern of systemic iron deficiency accompanied by focal iron accumulation within the joint. In RA synovial fluid, increased iron levels correlate with disease severity and are associated with enhanced lipid peroxidation in monocytes/macrophages. Moreover, macrophage subsets show heterogeneous susceptibility to iron-induced ferroptosis, partly related to differences in GPX4 regulation, suggesting that ferroptotic vulnerability may reshape the inflammatory macrophage compartment within the RA synovium (139). Thus, lipid peroxidation in RA should be viewed not only as an upstream signal that facilitates inflammasome activation but also as a mechanistic intersection with ferroptotic cell death.

### 6.3 Downstream inflammatory outputs: IL-1β, IL-18, pyroptosis, and intersecting cell-death programs

The main pathological consequences of NLRP3 inflammasome activation are maturation and release of IL-1β and IL-18 and GSDMD-mediated pyroptosis. IL-1β is an important effector cytokine in RA synovial inflammation and tissue destruction. It promotes RA-FLS activation, induces the expression of matrix metalloproteinases (MMPs), chemokines, and adhesion molecules, and enhances the inflammatory amplification and cartilage-invading capacity of FLS (116). IL-1β can also act on chondrocytes and bone-related cells, promoting cartilage matrix degradation and osteoclastogenic signals, thereby driving structural joint damage.

IL-18 is more closely associated with inflammatory T-cell responses, IFN-γ-related immune responses, and local synovial immune amplification. After mature IL-18 is released, it can enhance the inflammatory activity of T cells, natural killer cells, and other immune cells and cooperate with cytokines such as IL-12 and IL-15 to promote Th1-like inflammatory responses (140). Although IL-18 has been less widely targeted than IL-1β in RA, as an inflammasome output product it reflects the conversion of macrophage metabolic stress into immune-inflammatory amplification.

Pyroptosis further amplifies this pathological process. Pyroptotic macrophages release mature IL-1β, IL-18, and multiple intracellular danger-associated molecules, promoting the continued activation of surrounding FLS, endothelial cells, and infiltrating immune cells (141). Pyroptosis may also increase the burden of cellular debris and damage signals in the local synovium, further activating surviving macrophages and other immune cells (110). The NLRP3 inflammasome is, therefore, not merely a proteolytic platform for inflammatory cytokine maturation; it is a central effector pathway that links lipid metabolic stress, organelle damage, cell death, and synovial inflammatory amplification.

Importantly, regulated cell death in RA macrophages is not restricted to canonical GSDMD-mediated pyroptosis. Recent evidence demonstrates that synovial macrophages can also undergo PANoptosis, an integrated inflammatory cell-death program involving coordinated components of pyroptosis, apoptosis, and necroptosis. In RA synovium, reparative MerTK^+^CD206^+^ macrophages were shown to undergo PANoptotic death driven by C1q-dependent activation of SARM1, NAD^+^ depletion, metabolic collapse, and formation of an NLRC5/NLRP12-containing PANoptosome (142). This process promoted the release of inflammatory mediators and contributed to persistent synovial inflammation. These findings indicate that macrophage death in RA should be viewed as a broader and context-dependent network of regulated cell-death programs rather than as a process mediated exclusively by the NLRP3–caspase-1–GSDMD pyroptotic axis.

Taken together, the pathogenic significance of the NLRP3 inflammasome in RA lies in its capacity to convert macrophage lipid metabolic stress into tissue-level inflammatory amplification. Caspase-1-dependent maturation of IL-1β and IL-18, together with GSDMD-mediated pyroptosis, reinforces macrophage–FLS crosstalk, immune-cell recruitment, and osteoclastogenic signaling. However, macrophage death in RA is not restricted to canonical pyroptosis, and emerging evidence indicates that PANoptosis and ferroptosis may intersect with inflammasome-associated inflammatory responses in a context-dependent manner. These interconnected processes collectively link macrophage metabolic dysregulation to persistent synovitis and structural joint damage. The magnitude and cellular specificity of this axis across distinct RA synovial macrophage subsets and disease pathotypes remain to be defined. **Figure 3** summarizes the major mechanisms by which lipid metabolic stress induces NLRP3 inflammasome activation in RA macrophages and illustrates the relationships among FASN-dependent lipid synthesis, NLRP3 palmitoylation, mature cytokine release, and potential therapeutic intervention nodes.

**Figure 3:**
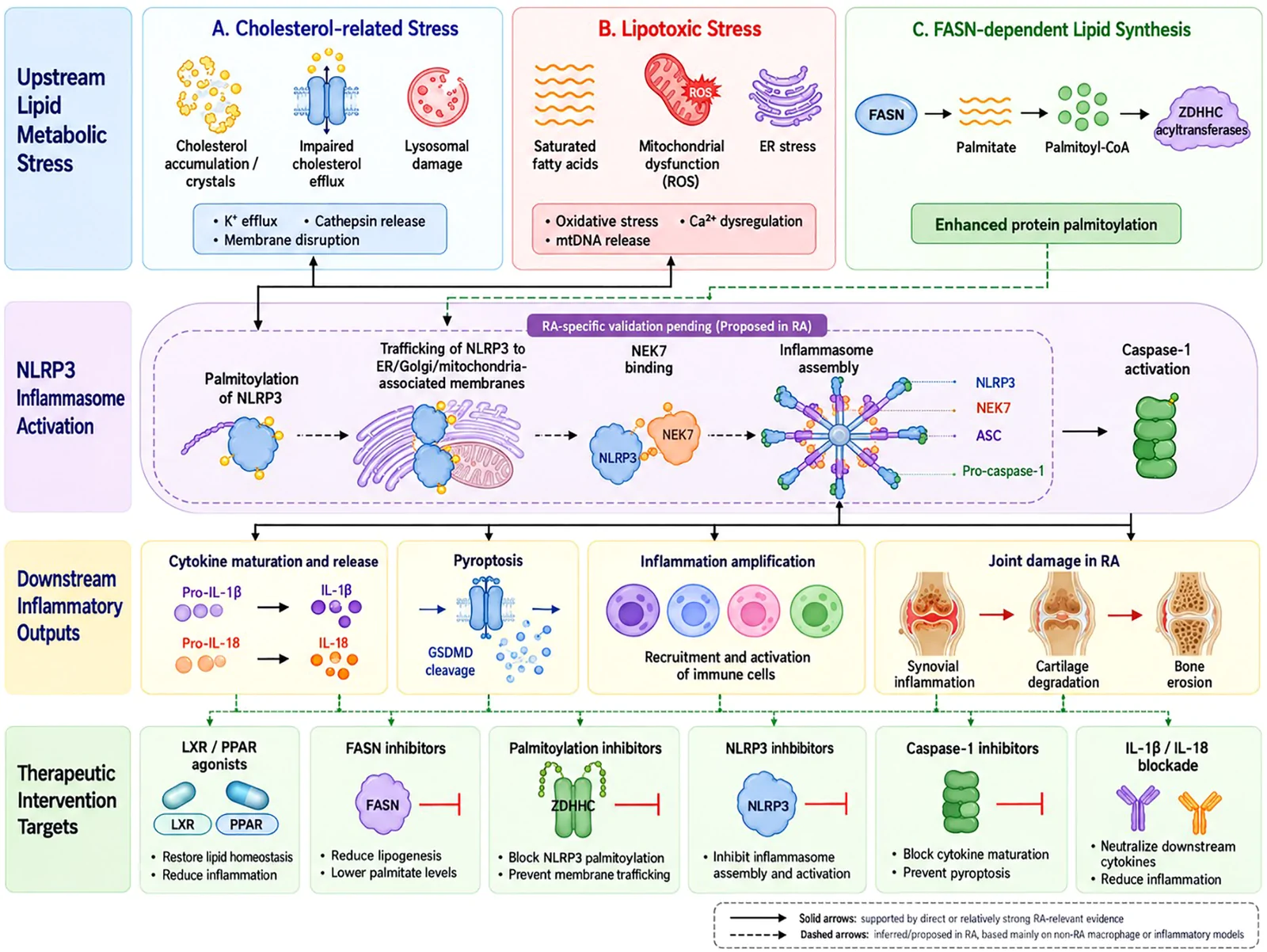
Lipid metabolic stress-driven NLRP3 inflammasome activation and potential therapeutic targets. **(A)** Cholesterol-related stress, including cholesterol accumulation or crystallization, impaired cholesterol efflux, and associated lysosomal damage. **(B)** Lipotoxic stress, including saturated fatty acid load, mitochondrial dysfunction, endoplasmic reticulum (ER) stress, and lipid peroxidation. **(C)** FASN-dependent lipid synthesis, in which FASN-derived palmitate can be converted into palmitoyl-CoA and provide acyl donors for protein S-palmitoylation. These lipid metabolic stresses promote NLRP3 inflammasome priming or activation through lysosomal damage, K+ efflux, mitochondrial reactive oxygen species (mtROS), mitochondrial DNA (mtDNA) release, ER stress, and related mechanisms. Evidence mainly from non-RA macrophage and inflammatory models suggests that NLRP3 palmitoylation may facilitate its subcellular trafficking, NEK7 binding, ASC recruitment, and inflammasome assembly, although this complete sequence remains to be directly validated in RA synovial macrophages. Activated caspase-1 promotes IL-1β and IL-18 maturation and GSDMD-mediated pyroptosis, thereby amplifying synovial inflammation and structural joint damage. Solid arrows indicate established or RA-supported relationships, whereas dashed arrows indicate mechanistic links proposed in RA but supported mainly by non-RA models.

## 7 Discussion

Macrophage lipid metabolic reprogramming in RA is better understood as a multilevel determinant of inflammasome competence than as a series of parallel metabolic alterations. Cholesterol-dependent membrane remodeling and inflammatory transcription primarily shape NLRP3 priming, whereas lipotoxicity and organelle stress provide activation-associated signals; FASN-dependent palmitoylation may further regulate the spatial trafficking and assembly of the inflammasome. These regulatory layers converge on caspase-1 activation, IL-1β/IL-18 maturation, and pyroptosis, thereby coupling macrophage-intrinsic metabolic disturbance to macrophage–FLS crosstalk, osteoclastogenic signaling, persistent synovitis, and structural joint damage. Within this hierarchy, LXR and PPAR function as complementary upstream lipid-sensing checkpoints, whereas the FASN–palmitoylation axis represents a mechanistically plausible bridge between lipid synthesis and inflammasome assembly. Importantly, the strength of evidence is uneven across this framework: RA-specific evidence is substantial for macrophage heterogeneity and inflammasome-associated inflammatory output, whereas the complete FASN–palmitoylation–NLRP3 sequence is supported mainly by mechanistic studies in non-RA macrophage or inflammatory models and remains to be directly validated in RA synovial macrophages. The pathogenic influence of macrophage lipid metabolism is, therefore, exerted primarily through modulation of macrophage responsiveness rather than through autonomous initiation of RA. By regulating membrane-receptor signaling thresholds, organelle stress, inflammasome competence, lipid-mediator output, and inflammation-resolution capacity, lipid metabolic programs determine how macrophages respond to autoantibodies, cytokines, and stromal signals and how effectively these responses are transmitted to FLS, lymphocytes, and osteoclast precursors. Its contribution is consequently expected to vary across synovial pathotypes and disease stages and should be regarded as a context-dependent regulatory layer within the broader RA inflammatory network rather than a universally dominant disease driver. The integrated framework linking the RA synovial microenvironment, macrophage lipid metabolic reprogramming, NLRP3 inflammasome regulation, and persistent synovial inflammation is summarized in **Figure 4**.

**Figure 4:**
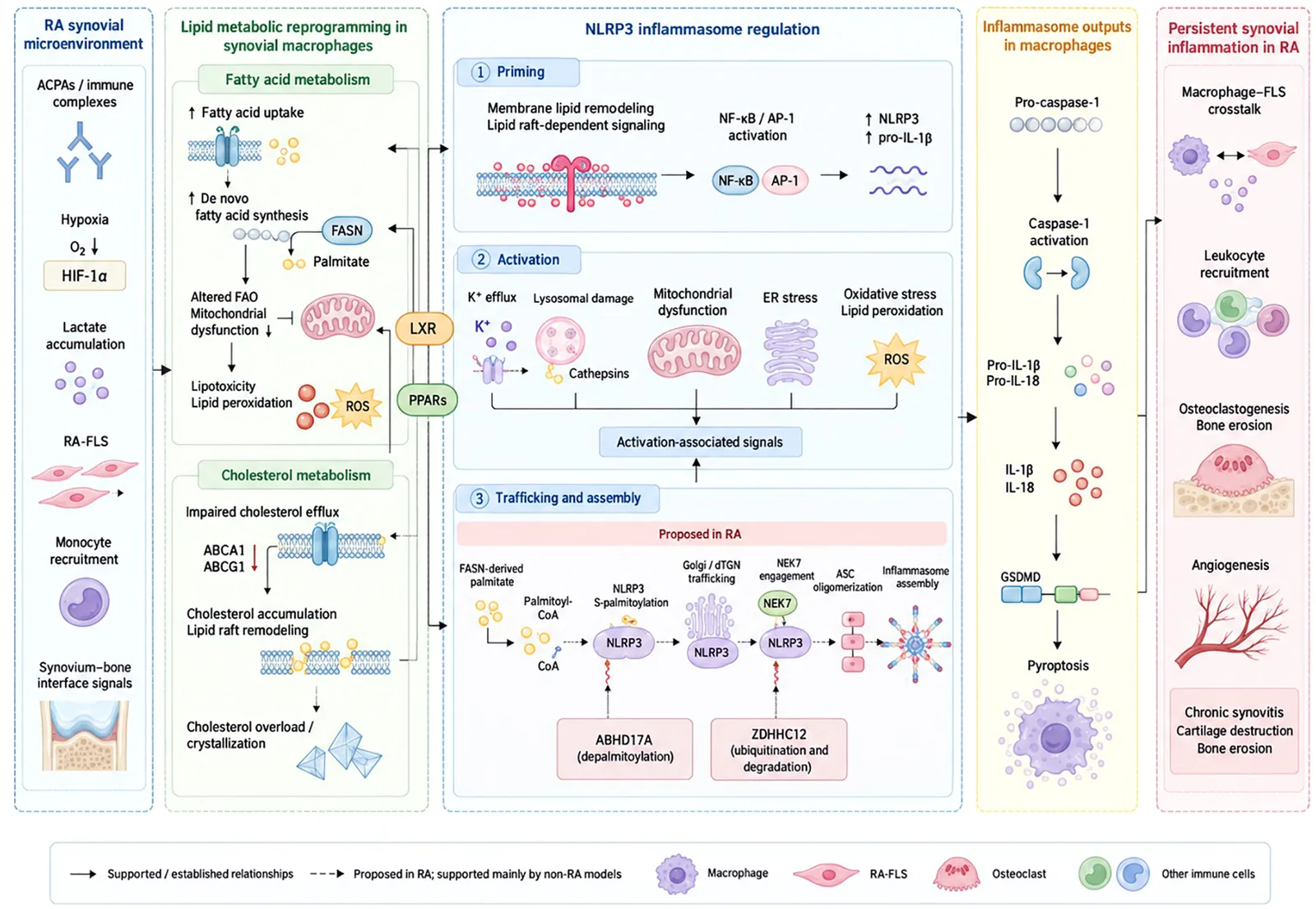
Integrated framework linking macrophage lipid metabolic reprogramming to NLRP3 inflammasome activation and persistent synovial inflammation in rheumatoid arthritis. The rheumatoid arthritis (RA) synovial microenvironment reshapes fatty acid and cholesterol metabolism in macrophages. Membrane lipid remodeling promotes NLRP3 priming, whereas lipotoxicity, organelle stress, oxidative stress, and ionic imbalance provide activation-associated signals. FASN-derived palmitate can be converted into palmitoyl-CoA, while mechanistic evidence mainly from non-RA models suggests that NLRP3 S-palmitoylation may facilitate Golgi/dTGN trafficking, NEK7 engagement, ASC oligomerization, and inflammasome assembly. Subsequent caspase-1 activation, IL-1β/IL-18 maturation, and GSDMD-mediated pyroptosis amplify macrophage–FLS crosstalk, immune-cell recruitment, osteoclastogenesis, and structural joint damage. Solid arrows indicate supported or established relationships, whereas dashed arrows indicate mechanistic links proposed in RA and supported mainly by non-RA models.

Patient-based evidence currently supports several components of this framework. Studies of human RA synovium and synovial fluid have identified heterogeneous macrophage states and altered inflammatory and pro-resolving lipid-mediator profiles, while patient-derived osteoclast precursors show CPT1A-dependent fatty acid oxidation associated with osteoclastogenesis (13, 67). However, most available studies are cross-sectional or involve relatively small cohorts, and direct measurements of lipid metabolic flux in RA synovial macrophages remain scarce. Clinical intervention studies of lipid-modifying therapies have included both PPARγ agonists and statins, although their therapeutic implications differ substantially. Although LXR and PPAR modulation is mechanistically attractive, their translation to RA therapy remains limited. Nonselective LXR agonism can activate hepatic lipogenic programs—for example, T0901317 induced marked hepatic steatosis and increased the secretion of triglyceride-rich very-low-density lipoprotein particles in experimental models, illustrating an on-target metabolic liability that narrows the therapeutic window of systemic LXR agonists (143). PPARγ agonists have reached small clinical studies in RA. In a randomized crossover trial, pioglitazone modestly reduced DAS28-CRP and CRP but did not significantly improve tender or swollen joint counts, while lower-extremity edema occurred more frequently than with placebo (144). Therefore, these agents should not be regarded as established RA therapies; future development will require receptor-selective or tissue-restricted modulation and clearer evidence of clinically meaningful joint-specific benefits.

Statins provide a distinct example of clinically available lipid-modifying therapy in RA. The TARA trial demonstrated modest reductions in disease activity and systemic inflammatory markers with atorvastatin, whereas the larger TRACE RA trial confirmed substantial LDL-cholesterol lowering and reduction of C-reactive protein but did not demonstrate a statistically significant reduction in the primary cardiovascular endpoint (63, 64). These findings suggest that statins may exert anti-inflammatory effects in RA, but their established clinical role remains cardiovascular risk reduction rather than disease-modifying antirheumatic therapy.

Beyond nuclear receptors, FASN and protein palmitoylation provide new approaches for targeting the link between lipid synthesis and inflammasome activation. FASN-mediated *de novo* fatty acid synthesis not only supplies substrates for membrane lipid renewal and organelle remodeling but may also increase palmitate and palmitoyl-CoA supply, promote palmitoylation of inflammation-related proteins such as NLRP3, and thereby affect subcellular localization, trafficking, and inflammasome assembly. In theory, inhibiting FASN activity, limiting palmitate supply, or regulating ZDHHC family palmitoyltransferase activity may all reduce NLRP3 inflammasome activation and IL-1β/IL-18 release. At present, however, direct evidence for the FASN-NLRP3 palmitoylation axis in RA remains insufficient and requires further validation in primary RA synovial macrophages, animal models, and clinical samples.

As a downstream effector pathway of lipid metabolic stress, the NLRP3 inflammasome also has clear therapeutic appeal. Metabolic abnormalities such as cholesterol accumulation, lipotoxic stress, mtROS, and lipid peroxidation can lower the NLRP3 activation threshold, making macrophages more prone to release mature IL-1β and IL-18 and undergo GSDMD-mediated pyroptosis. Targeting NLRP3, caspase-1, GSDMD, or the IL-1β/IL-18 axis may, therefore, help block the conversion of lipid metabolic disturbance into cytokine release. RA, however, is not a disease driven by a single inflammasome pathway. Clinical experience with IL-1 blockade provides an important constraint on interpreting the NLRP3–IL-1 axis as a dominant therapeutic bottleneck in RA. Anakinra, an IL-1 receptor antagonist approved for moderately to severely active RA after inadequate response to one or more DMARDs, produces significant but generally modest clinical responses (145). Randomized trials and systematic reviews have shown improvements in ACR response rates compared with placebo, but the magnitude of benefit is relatively limited compared with that reported for other biologic therapies (146). The limited role of IL-1 blockade in contemporary RA treatment strategies, therefore, indicates that IL-1 signaling is unlikely to represent a universal disease-driving node. The NLRP3–IL-1β axis may be better viewed instead as a context-dependent inflammatory amplifier whose contribution varies according to synovial pathotype, macrophage state, and disease stage. Parallel TNF-α, IL-6/JAK, adaptive immune, fibroblast, and osteoclastogenic pathways may sustain disease activity even when IL-1 signaling is inhibited. Regulated cell death in RA macrophages likewise extends beyond canonical GSDMD-mediated pyroptosis. Emerging evidence indicates that PANoptosis and ferroptosis can also contribute to macrophage dysfunction and inflammatory amplification in the RA synovium, highlighting substantial crosstalk among inflammasome signaling, metabolic stress, lipid peroxidation, and distinct cell-death programs (139, 142). Its pathology also involves autoantibodies, lymphocyte responses, FLS invasion, osteoclast differentiation, and multiple cytokine networks. Blocking NLRP3 alone may be insufficient to fully control disease activity. A more rational strategy may combine inflammasome inhibition with metabolic intervention, disease-modifying antirheumatic drugs (DMARDs), biologics, or JAK inhibitors. These approaches may act at partially complementary levels. Biologics targeting inflammatory cytokines and JAK inhibitors primarily suppress cytokine-driven NF-κB/STAT signaling and inflammatory gene expression, thereby potentially reducing inflammasome priming, whereas lipid metabolism-targeting strategies may additionally modify membrane organization, organelle stress, and NLRP3 activation or assembly. JAK inhibition has also been shown to suppress inflammatory responses in RA synovial macrophages and to affect metabolically defined pathogenic CD14+ cell populations. However, direct clinical evidence supporting combinations of lipid metabolism-targeting agents with biologics or JAK inhibitors remains unavailable, and potential benefits must be balanced against overlapping immunosuppression, infection risk, and systemic metabolic toxicity (147, 148). Accordingly, interventions targeting macrophage lipid metabolism are more likely to be effective as biomarker-guided, macrophage-subset-selective, or combination strategies than as universally applicable stand-alone therapies.

Clinical translation of FASN and NLRP3 inhibition remains at an earlier stage. FASN inhibitors have demonstrated target engagement in early oncology trials and histological efficacy in a phase 2b trial of metabolic dysfunction-associated steatohepatitis; however, reversible dermatological and ocular toxicities and an increased incidence of alopecia highlight the difficulty of systemically inhibiting a fundamental lipid-synthetic enzyme (149, 150). Direct NLRP3 inhibitors have shown clinical proof of concept in gout and other inflammasome-mediated disorders, whereas their therapeutic efficacy in RA has not been established, and current RA evidence remains predominantly preclinical (151). This translational gap may reflect not only insufficient disease-specific validation but also the pathway redundancy of RA, in which TNF-α, IL-6, JAK signaling, autoantibodies, FLS invasion, and osteoclastogenesis may sustain disease activity despite inflammasome inhibition. Future development should, therefore, prioritize synovium- or macrophage-selective delivery using concrete approaches such as intra-articular sustained-release formulations, folate receptor-β (FRβ)-targeted nanocarriers for activated synovial macrophages, or mannose/CD206-directed liposomal or polymeric nanoparticles (152, 153). These strategies may enhance local drug exposure while limiting systemic metabolic toxicity. However, macrophage-targeting receptors are heterogeneously expressed across synovial subsets; therefore, receptor-directed delivery, particularly CD206-based approaches, requires subset-specific validation before clinical translation. Biomarker-based identification of patients with high inflammasome activity and combination with established DMARDs may further improve therapeutic selectivity and efficacy.

Based on the mechanisms described above, interventions targeting macrophage lipid metabolism in RA can be developed at multiple levels, including nuclear receptor regulation, lipid synthesis and modification, inflammasome output, and downstream cytokine blockade. Potential targets, therapeutic value, and translational challenges are summarized in **Table 3**.

**Table 3:** Potential therapeutic targets related to macrophage lipid metabolism in RA and translational challenges.

Target/strategy	Main point of action	Potential therapeutic value	Major limitations	Future research directions	References
LXR modulators	Regulate ABCA1/ABCG1-mediated cholesterol efflux, membrane cholesterol composition, and inflammatory transcriptional responses	Improve cholesterol homeostasis and modulate TLR sensitivity and glycolysis-associated inflammatory responses	Systemic LXR activation may induce hepatic lipogenesis, steatosis, and hypertriglyceridemia, thereby narrowing the therapeutic window; clinical evidence in RA remains lacking	Develop synovium- or macrophage-selective LXR modulation strategies	(154)
PPARγ modulators	Regulate fatty acid uptake, lipid storage, anti-inflammatory transcription, and autophagy-related ROS clearance	Suppress NF-κB-related inflammatory responses and promote inflammation-resolving and tissue repair-associated macrophage states	A small RA trial showed only modest reductions in DAS28-CRP and CRP, without significant improvement in tender or swollen joint counts; edema, weight gain, and other systemic metabolic effects limit routine clinical use	Clarify differences in responses to PPARγ modulation among RA macrophage subsets	(155)
Statins	Inhibit HMG-CoA reductase and the mevalonate pathway, reducing cholesterol synthesis and potentially modifying membrane lipid organization and inflammatory signaling	Lower LDL cholesterol and cardiovascular risk; may modestly reduce systemic inflammatory activity and RA disease activity	Not established as DMARDs for RA; anti-inflammatory effects are modest and cardiovascular outcome benefit in RA has not been definitively demonstrated	Define whether specific RA subgroups derive joint-specific anti-inflammatory benefit beyond cardiovascular risk reduction	(63, 64)
PPARα/PPARδ modulators	Promote FAO, peroxisomal lipid metabolism, and mitochondrial energy homeostasis	Reduce lipotoxic pressure and improve macrophage metabolic adaptation and inflammation resolution capacity	Enhanced FAO is not anti-inflammatory in all inflammatory contexts; effects depend on mitochondrial state and lipid load	Use metabolic flux analysis to clarify their true functions in RA synovial macrophages	(49)
FASN inhibition	Reduce *de novo* fatty acid synthesis and the supply of palmitate and palmitoyl-CoA	May reduce membrane lipid remodeling, TLR signaling platforms, and NLRP3 inflammasome assembly	Early clinical studies outside RA have reported dermatological and ocular toxicities and alopecia, reflecting the limited tissue selectivity of systemic FASN inhibition; no clinical evidence is currently available in RA	Validate efficacy and safety in primary RA synovial macrophages and animal models	(10)
ZDHHC- and ABHD17A-mediated palmitoylation regulation	Regulate site-specific NLRP3 palmitoylation and depalmitoylation, thereby influencing membrane trafficking, NEK7 interaction, ASC oligomerization, and NLRP3 degradation	May disrupt pro-activating NLRP3 palmitoylation or enhance negative regulatory pathways that limit sustained inflammasome signaling	ZDHHC enzymes and depalmitoylases have broad substrate repertoires, and different NLRP3 palmitoylation events may exert opposing effects; enzyme, modification site, and tissue selectivity, therefore, remain major challenges	Define the key ZDHHC enzymes, depalmitoylases, and NLRP3 modification sites in RA synovial macrophages and determine whether individual palmitoylation events promote inflammasome assembly or NLRP3 degradation	(48)
NLRP3 inflammasome inhibition	Block NLRP3 assembly, ASC recruitment, and caspase-1 activation	Reduce IL-1β and IL-18 maturation/release and pyroptosis-associated inflammatory amplification	RA is not driven by NLRP3 alone; monotherapy may be insufficient to control global inflammation	Evaluate in combination with lipid metabolic interventions, DMARDs, biologics, or JAK inhibitors	(28)
Caspase-1/GSDMD inhibition	Block IL-1β/IL-18 maturation and execution of pyroptosis	May reduce cytokine release, leakage of cellular contents, and spread of synovial inflammation	May affect host anti-infective defense and normal inflammatory clearance	Define molecular subtypes of RA patients with high pyroptotic activity	(156)
IL-1β/IL-18 blockade	Block mature inflammasome-derived cytokines downstream of NLRP3 signaling	Reduce inflammatory amplification, immune-cell recruitment, cartilage matrix degradation, and Th1-like inflammatory responses	Clinical efficacy of IL-1 blockade in RA is generally modest; anakinra has a limited role in contemporary RA treatment, indicating that IL-1 signaling is not a universal therapeutic bottleneck. Blocking IL-1β/IL-18 does not address parallel TNF-α, IL-6/JAK, adaptive immune, fibroblast, and osteoclastogenic pathways	Identify synovial pathotypes or biomarker-defined patient subsets with high NLRP3/IL-1β/IL-18 activity that may preferentially benefit from pathway-directed therapy	(116, 145)
SPM supplementation or mimetics	Enhance signaling by SPMs such as resolvins, protectins, and maresins	Promote efferocytosis, tissue repair, and termination of inflammation	*In vivo* stability, delivery route, dosing, and clinical efficacy in RA require further clarification	Develop local joint delivery and SPM-response-based patient stratification strategies	(73, 87)

Future studies should move beyond descriptive profiling and determine whether distinct RA synovial macrophage subsets possess stable and functionally relevant lipid-metabolic programs. Key questions include whether differences in fatty acid uptake, *de novo* lipogenesis, fatty acid oxidation, cholesterol efflux, lipid mediator production, and NLRP3 susceptibility are causes or consequences of macrophage inflammatory states and whether these metabolic features predict disease activity or therapeutic response. Single-cell metabolomics may help identify subset-specific metabolic states that cannot be inferred reliably from transcriptomic data alone, whereas spatial metabolomics may determine whether these states are preferentially localized in the synovial lining, perivascular regions, macrophage–FLS niches, or the synovium–bone interface. Integration of single-cell metabolomics with single-cell RNA sequencing, single-cell ATAC sequencing, spatial transcriptomics, synovial lipidomics, isotope-based metabolic flux analysis, and functional perturbation will be required to connect macrophage identity, anatomical location, metabolic activity, inflammasome responsiveness, and tissue damage. Longitudinal analysis of patient samples before and after treatment may further identify metabolic biomarkers that support patient stratification and prediction of therapeutic response.

Taken together, macrophage lipid metabolic reprogramming represents a modifiable regulator of inflammasome responsiveness rather than an independent driver of RA. Future studies should define metabolically distinct synovial macrophage subsets, validate lipid-related biomarkers for patient stratification, and determine whether selective correction of lipid dysregulation can improve the efficacy of inflammasome inhibition or established DMARDs. Clinical translation will ultimately require practical synovium- or macrophage-targeted delivery strategies, including intra-articular sustained-release systems and receptor-directed nanocarriers, together with subset-specific validation in longitudinal human cohorts and mechanism-based intervention trials.
